# Supplementary material for: Stratigraphic architecture of the Belly River Group (Campanian, Cretaceous) in the plains of southern Alberta: Revisions and updates to an existing model and implications for correlating dinosaur-rich strata
Source: PLoS One. 2024 Jan 25;19(1):e0292318. doi: 10.1371/journal.pone.0292318 (PMC10810474; doi:10.1371/journal.pone.0292318)

#01
06-36-01-30W3 SK

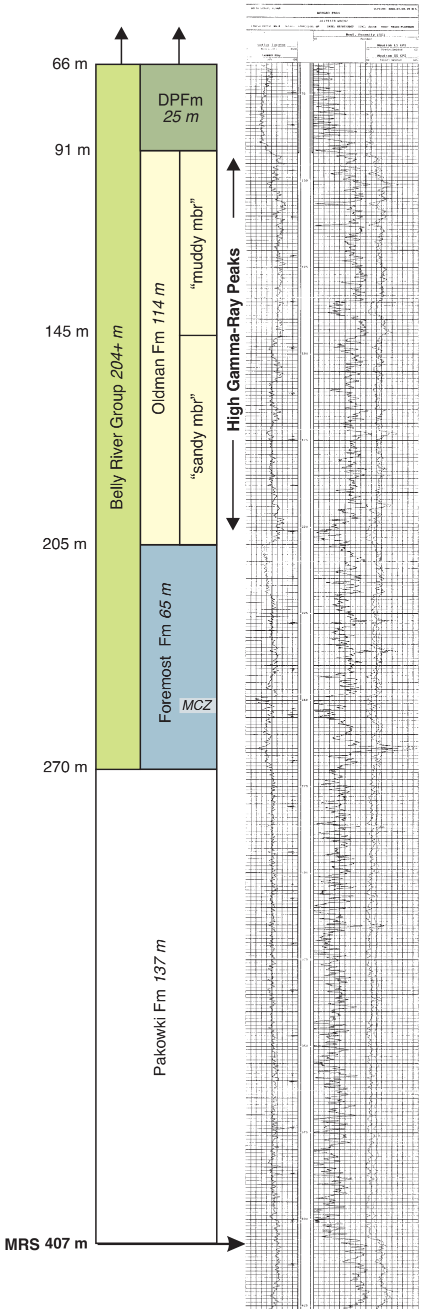

#02
Reference well
14-14-02-30W3

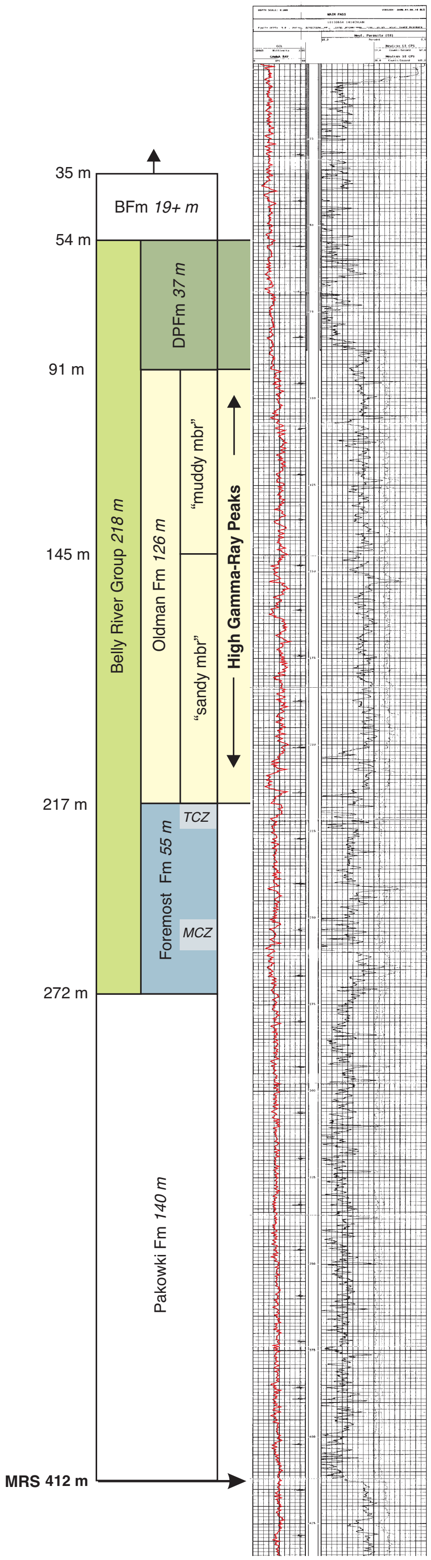

#03
Reference well
08-36-03-30W3

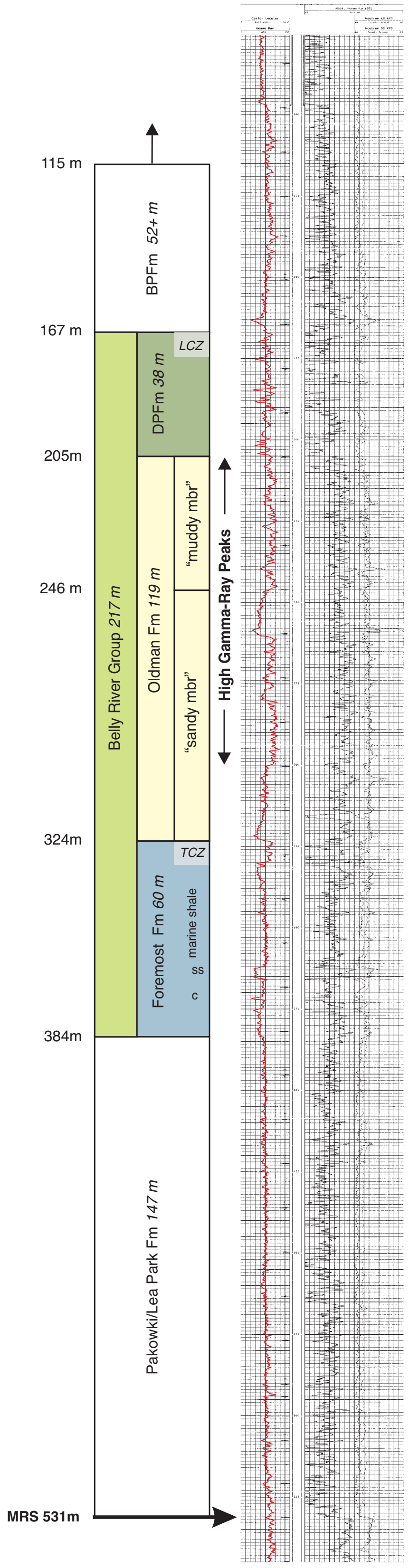

#04
Reference well
11-25-04-01W4

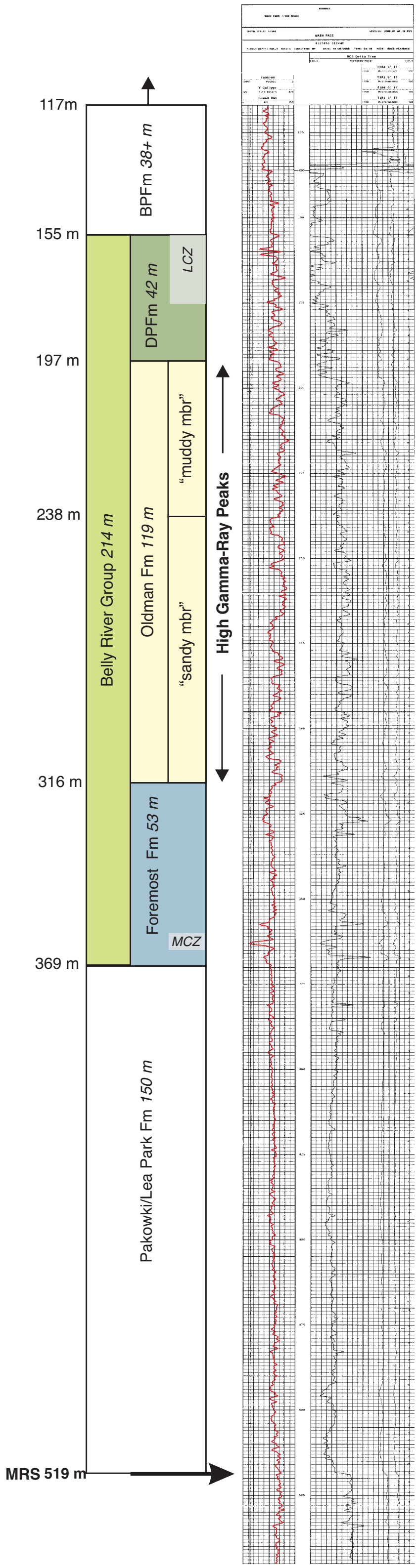

#05
04-27-05-02W4

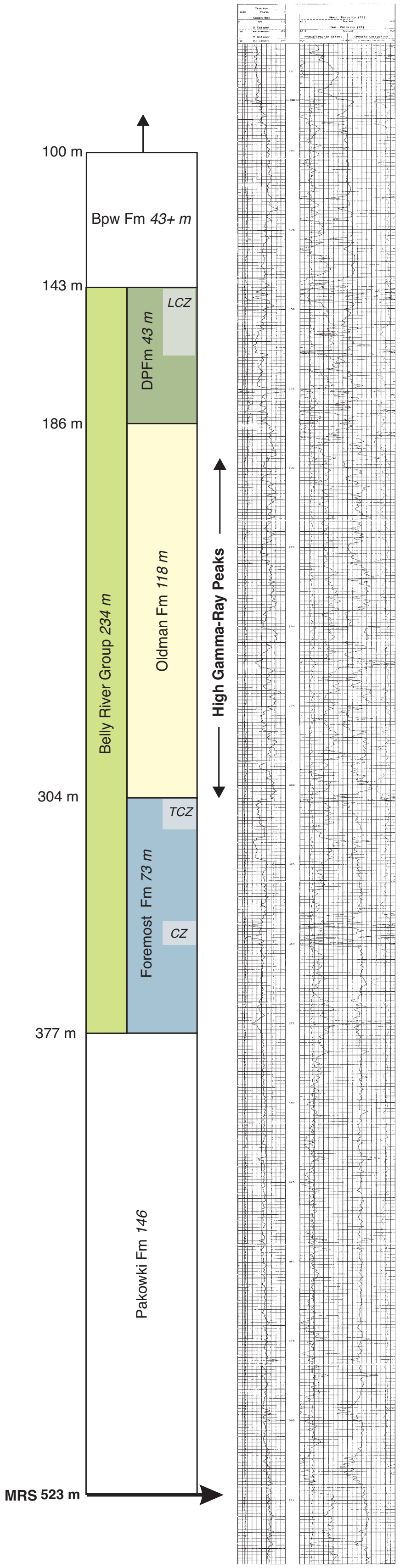

#06
05-35-06-02W4

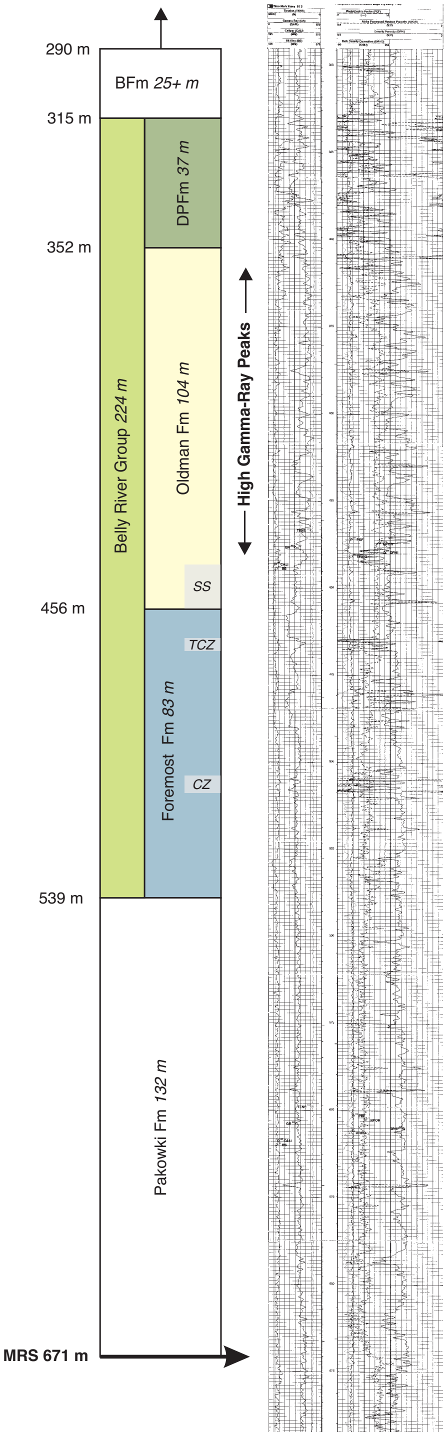

#07
03-16-07-02W4

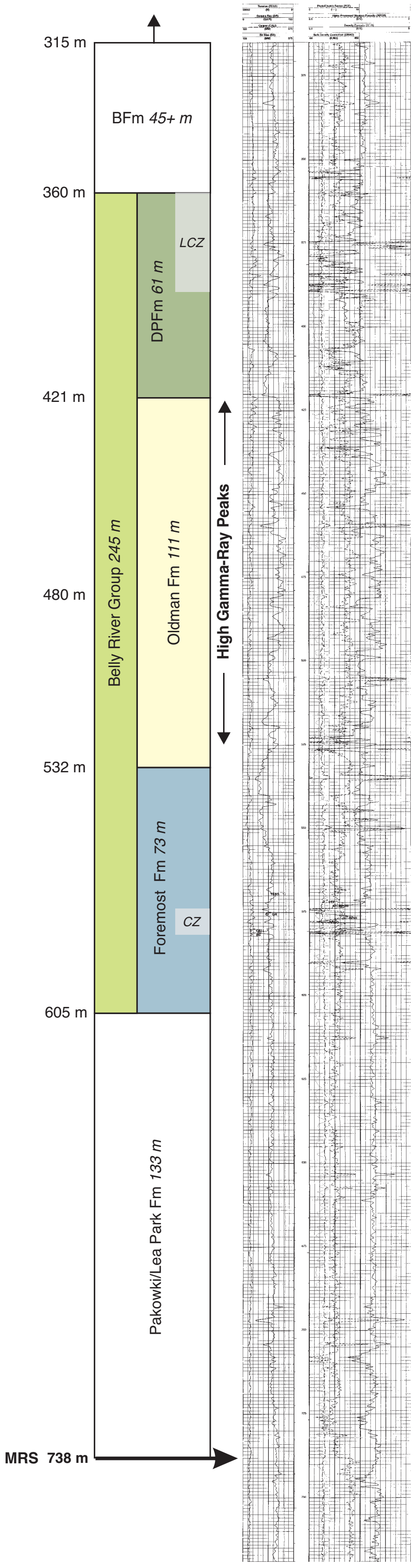

#08
04-32-08-03W4

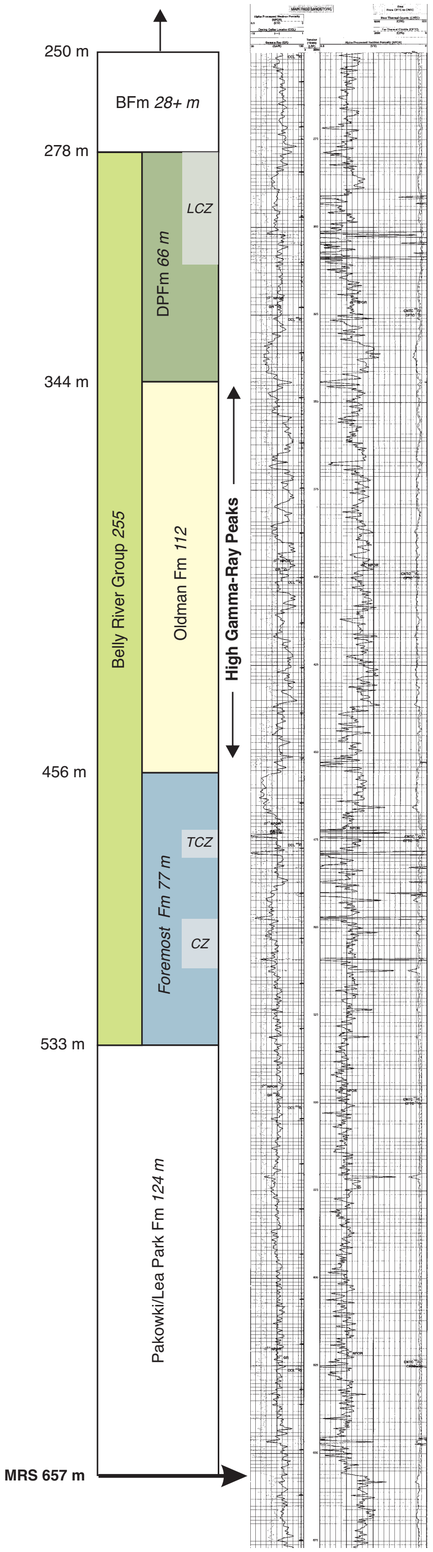

#09
06-14-09-03W4

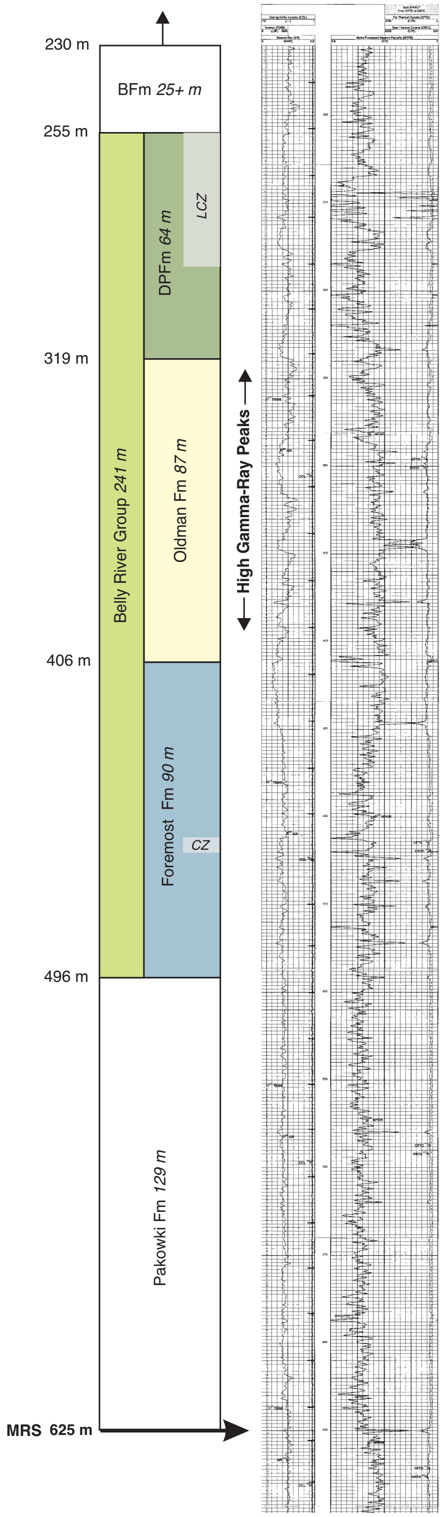

#10
06-06-10-03W4

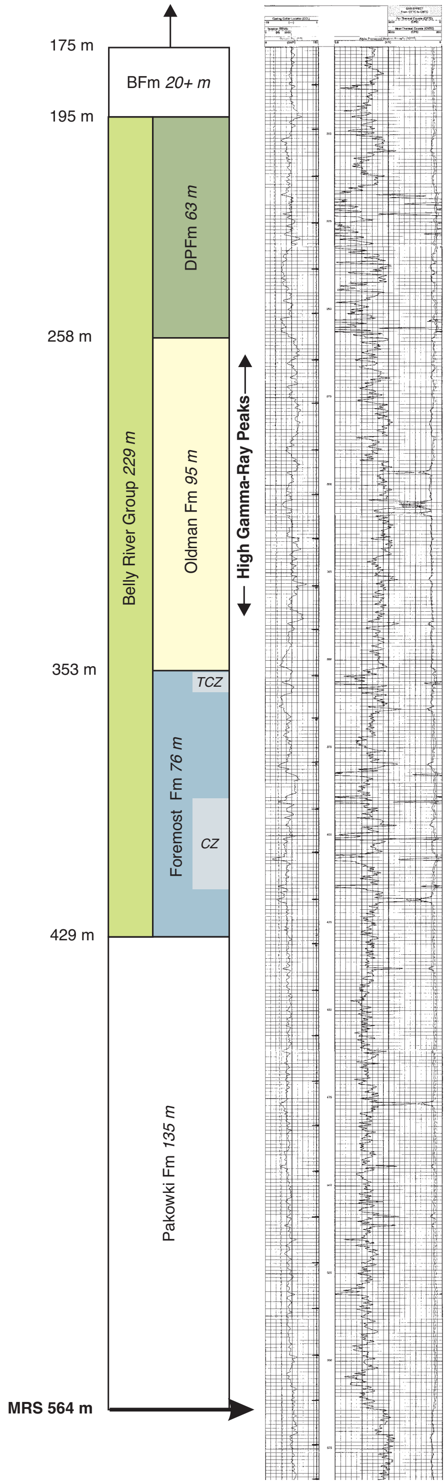

#11
16-04-11-03W4

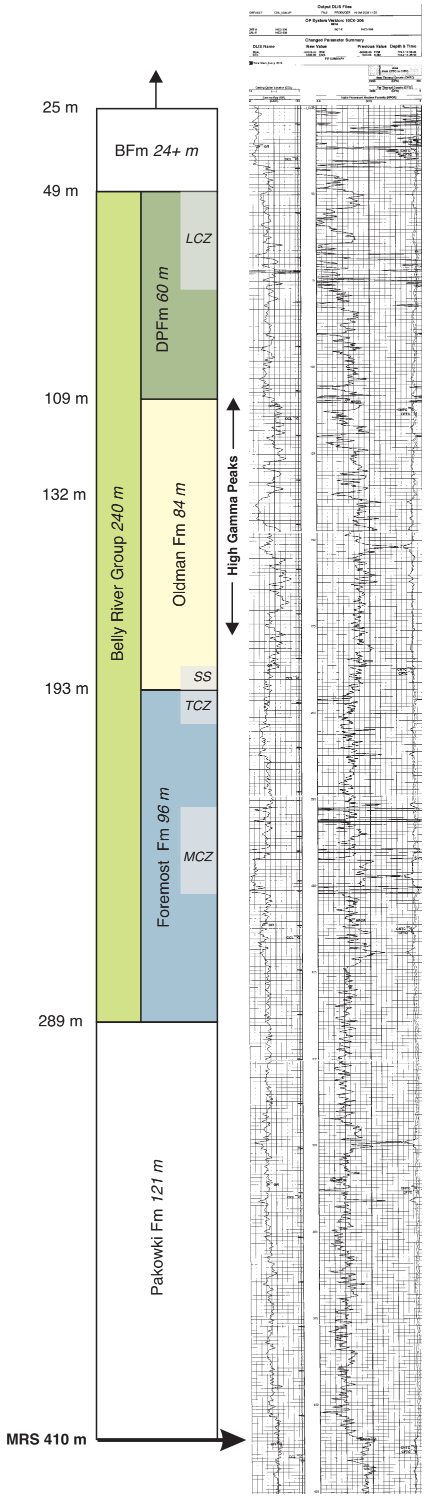

#12
09-13-12-03W4

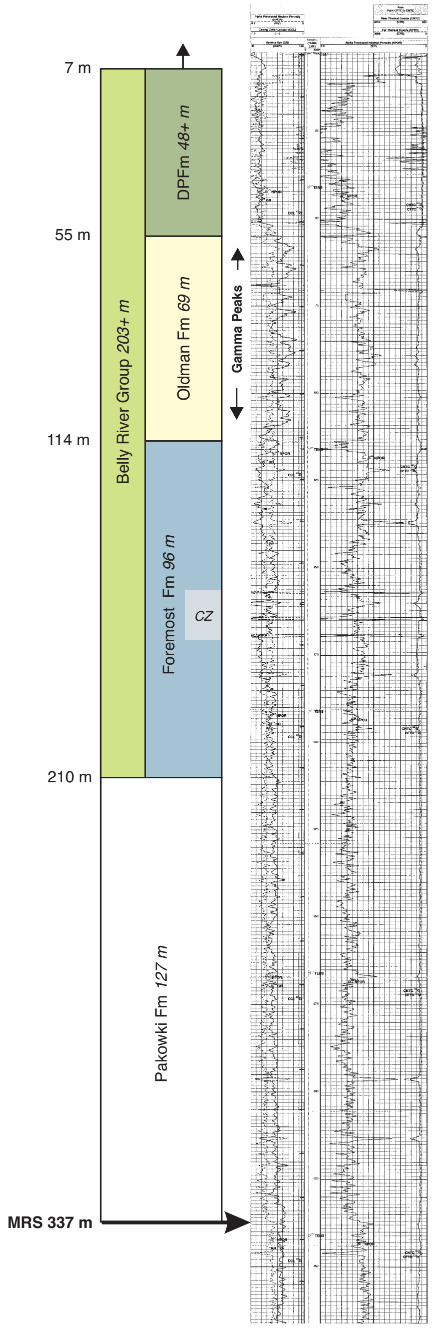

#13
14-06-13-03W4

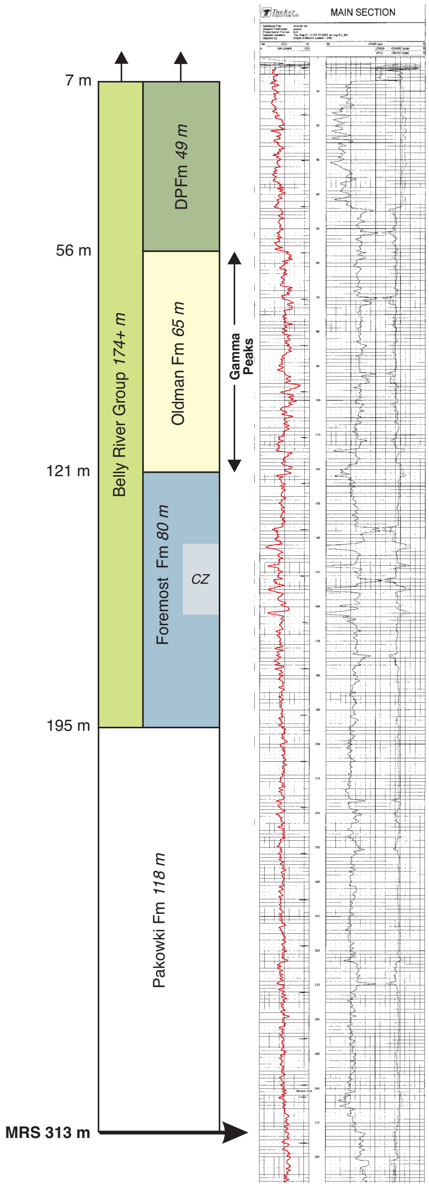

#14
12-19-14-02W4

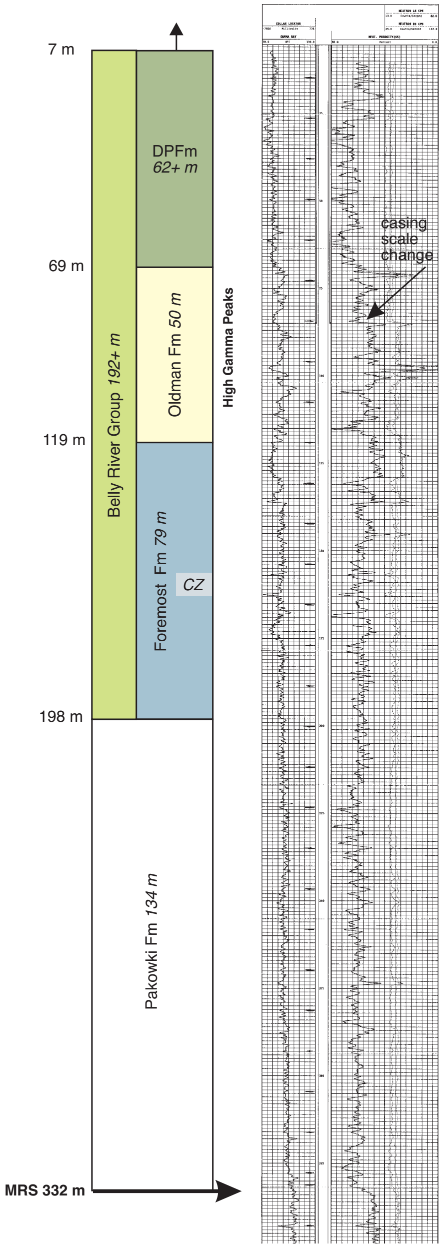

#15
11-14-15-03W4

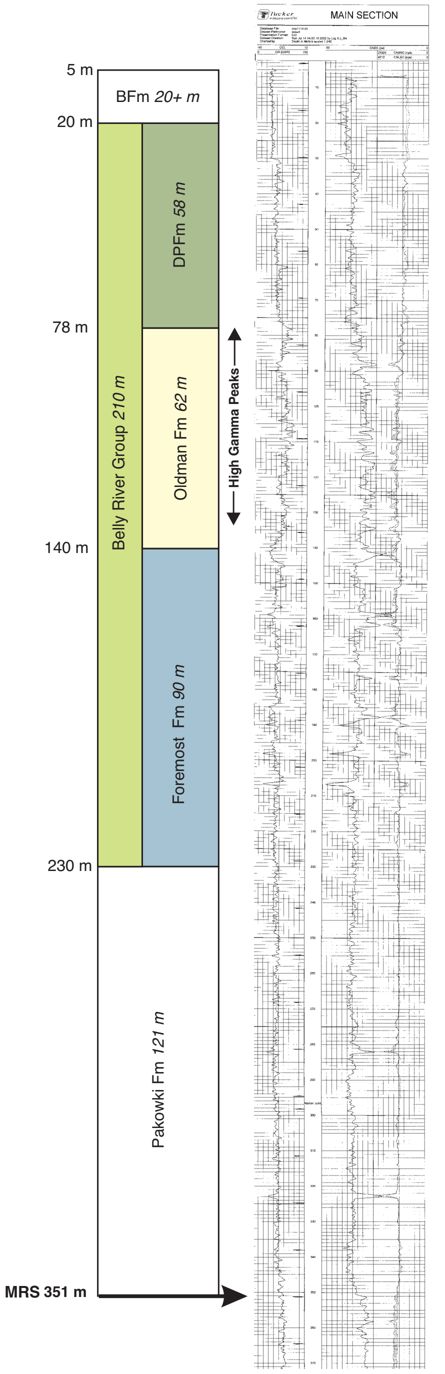

#16
14-22-16-02W4

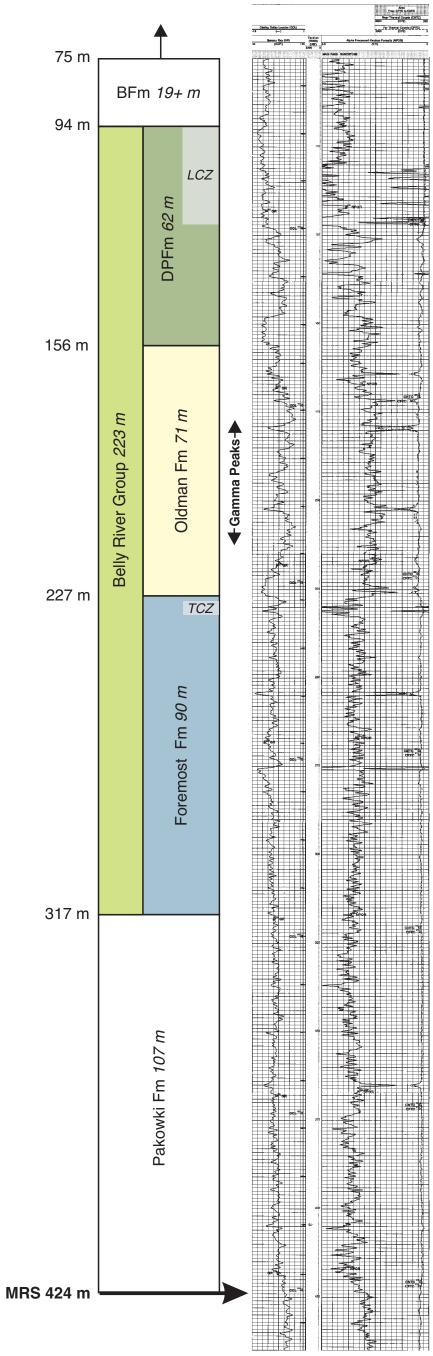

#17
Reference well
16-15-17-02W4

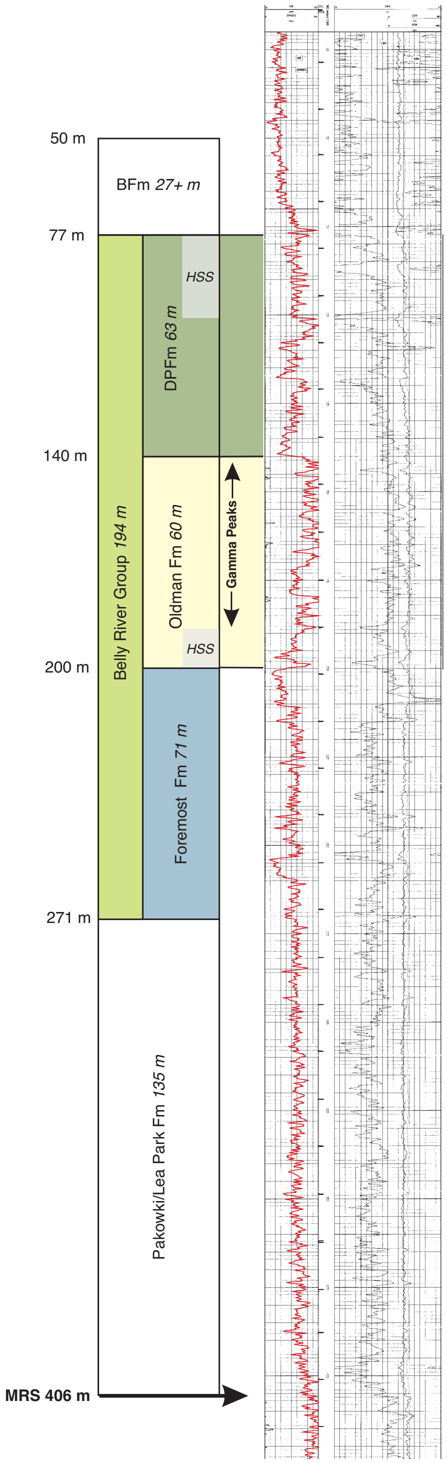

#18
04-04-18-02W4

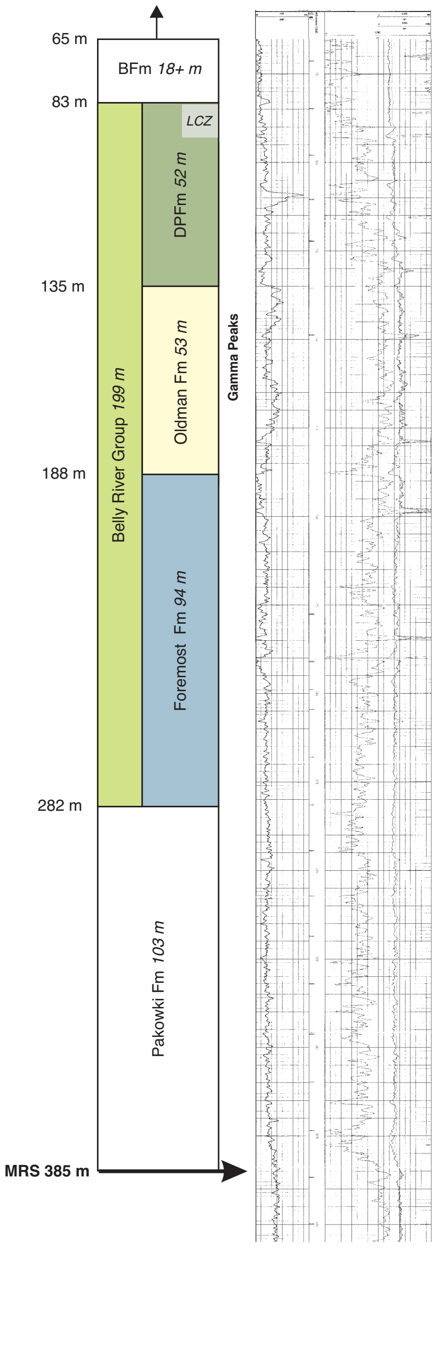

#19
12-24-19-29W3

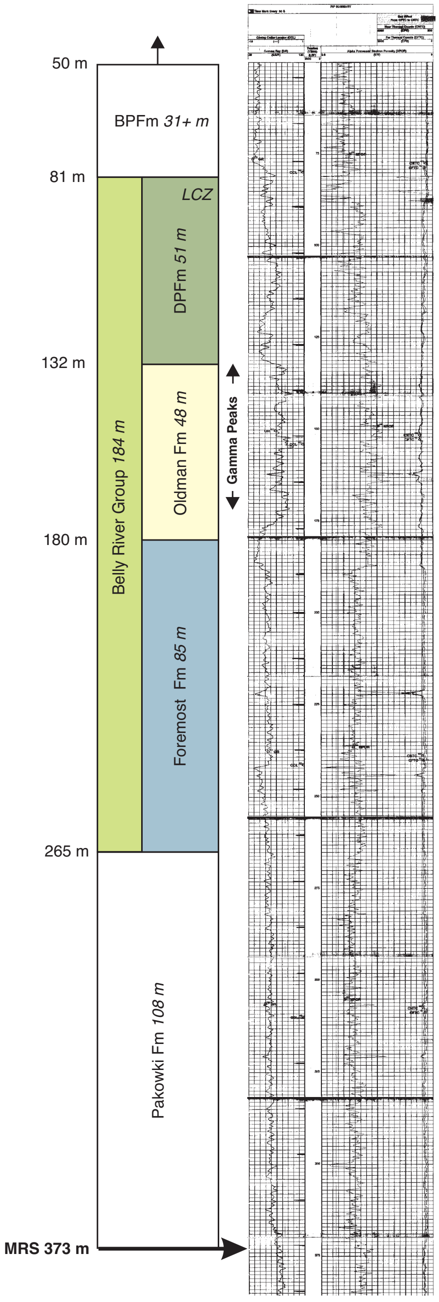

#20
16-04-20-29W3

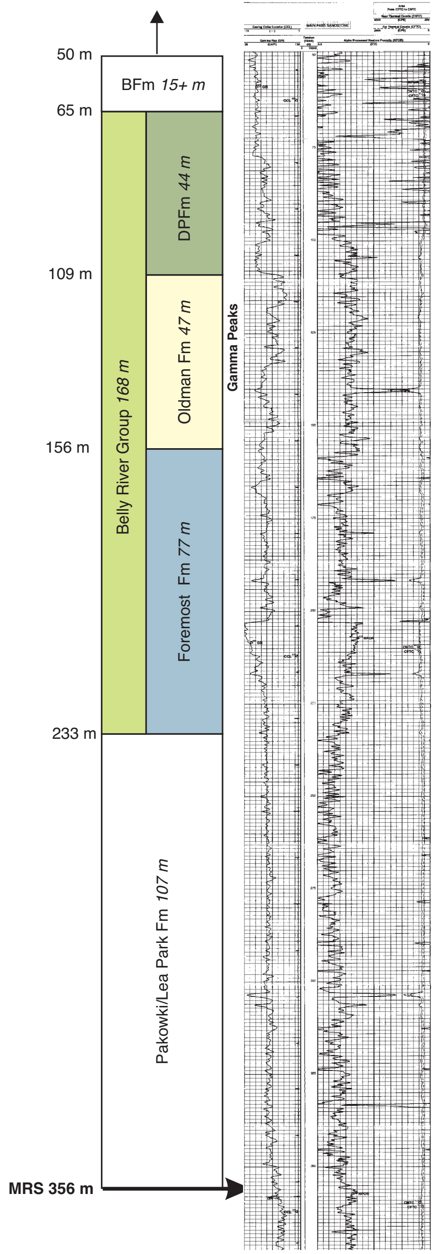

#21
Reference well
06-12-21-01W4

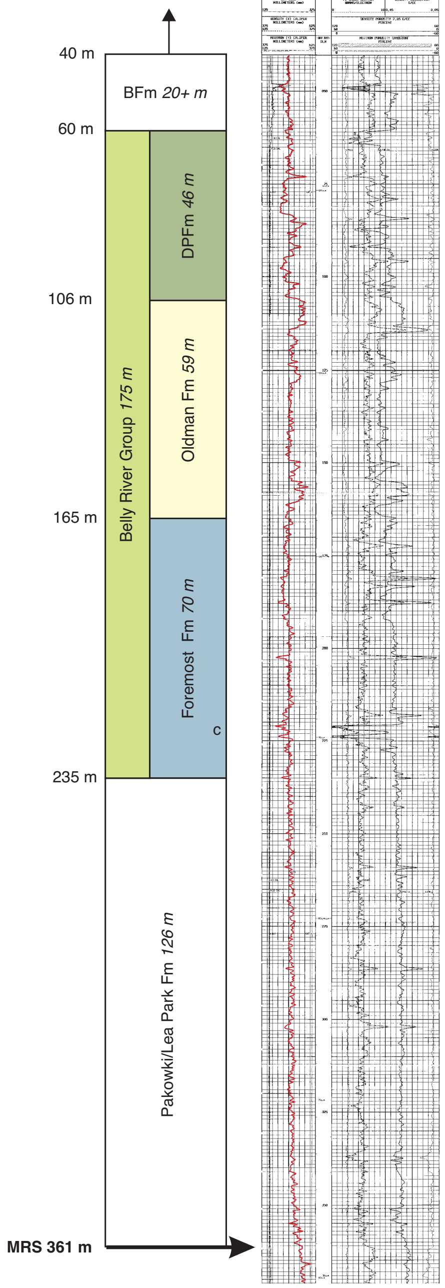

#22
03-02-22-02W4

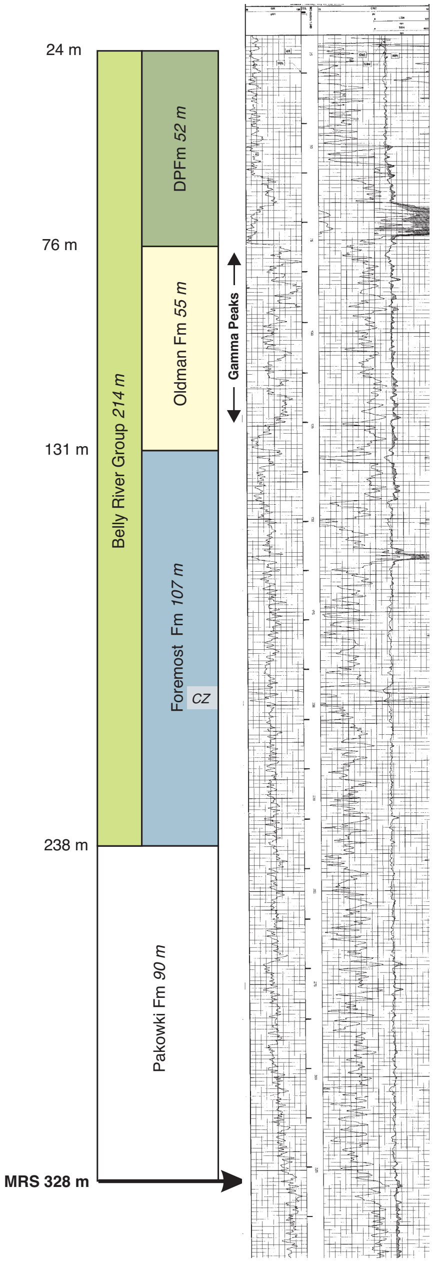

#23
14-31-23-03W4

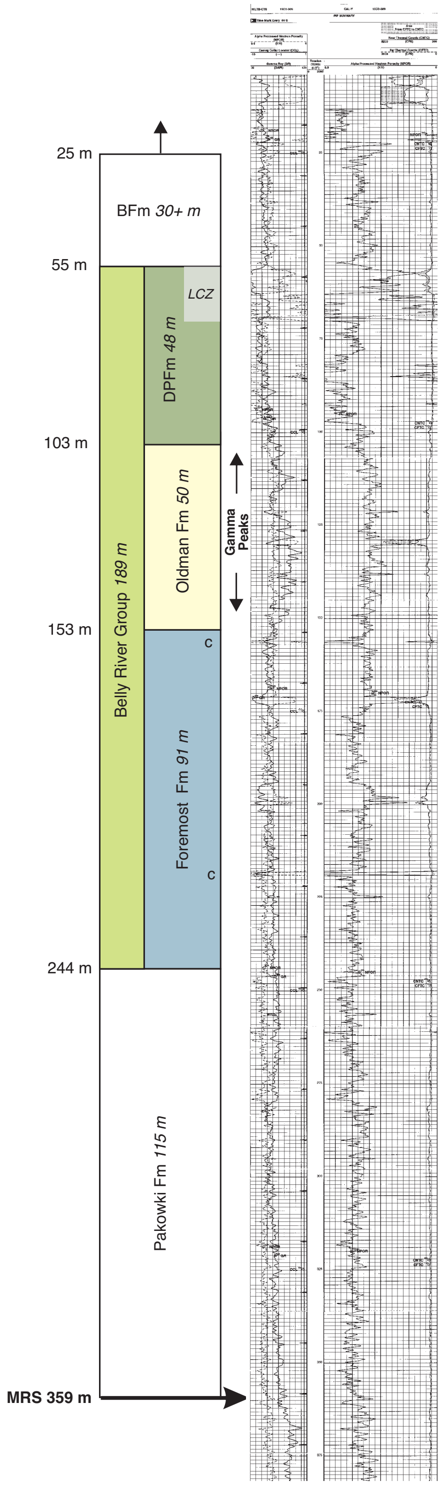

#24
12-20-24-02W4

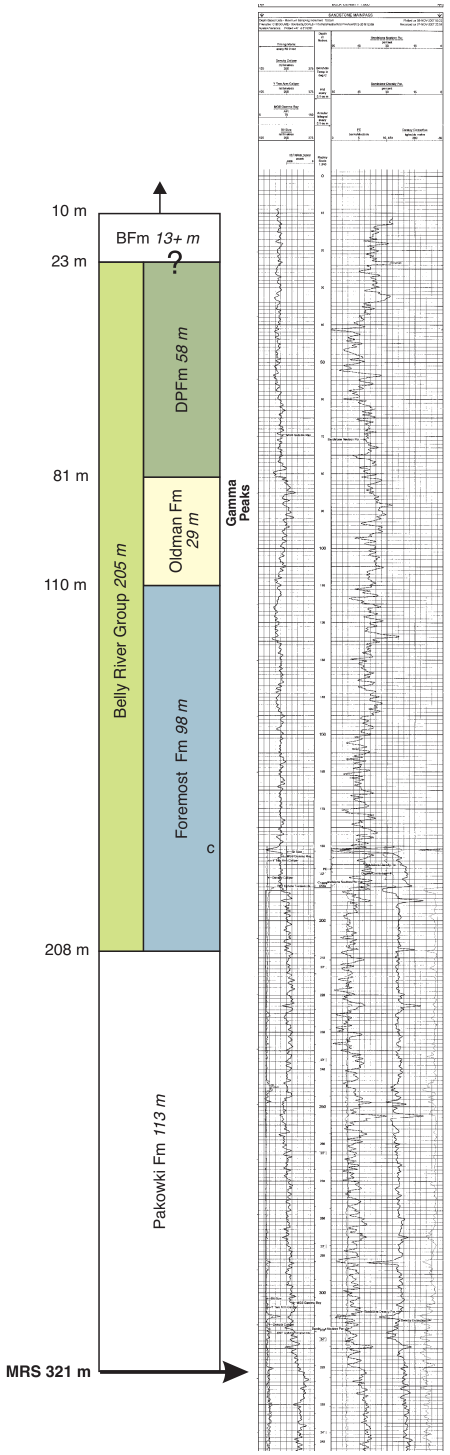

#25
16-30-25-02W4

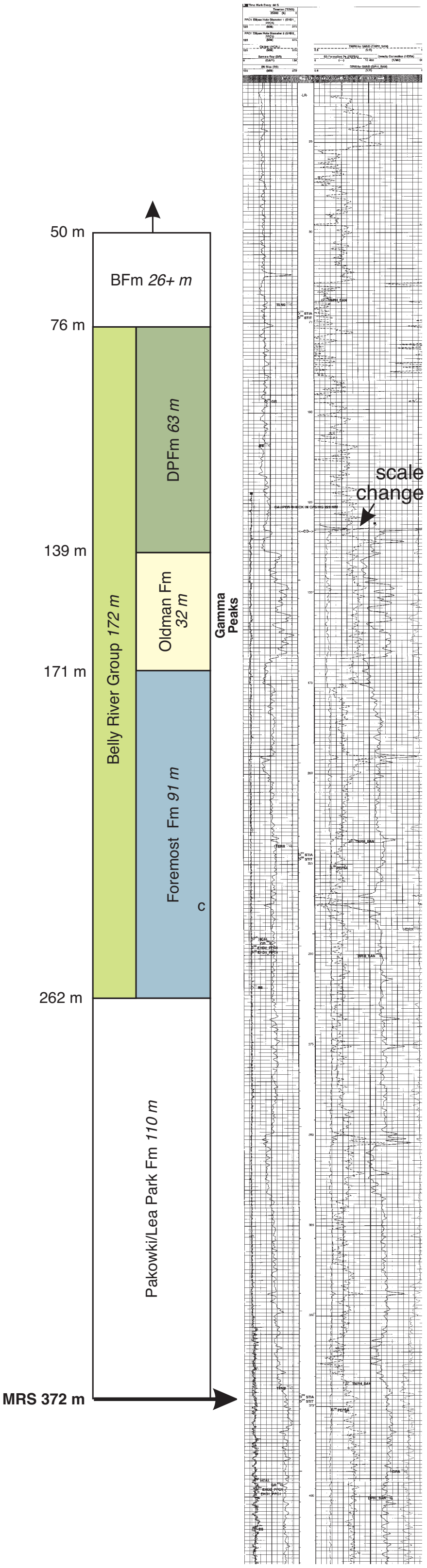

#26
11-30-26-03W4

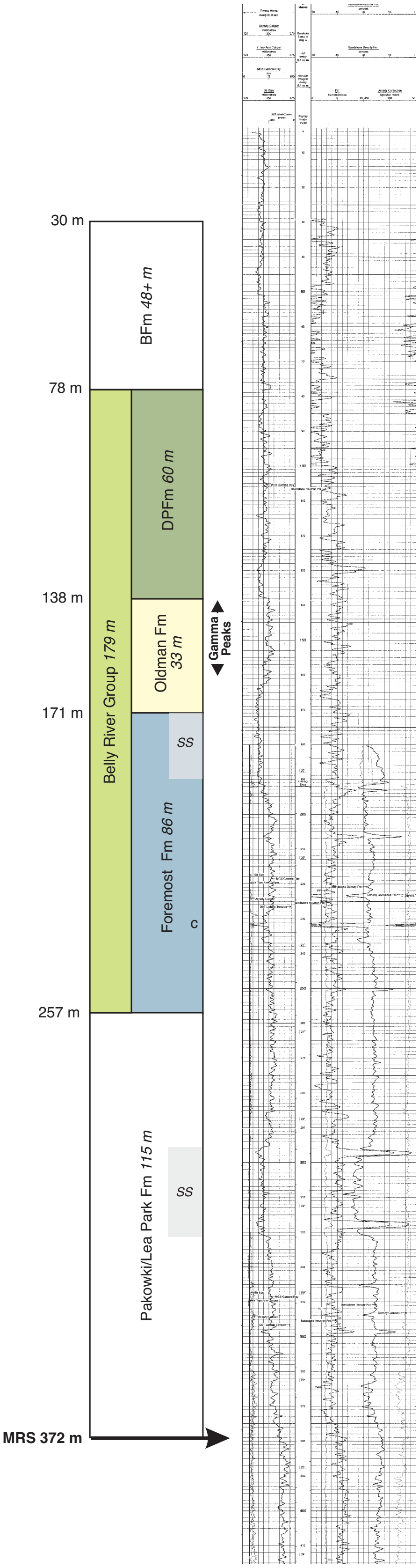

#27
15-21-27-03W4

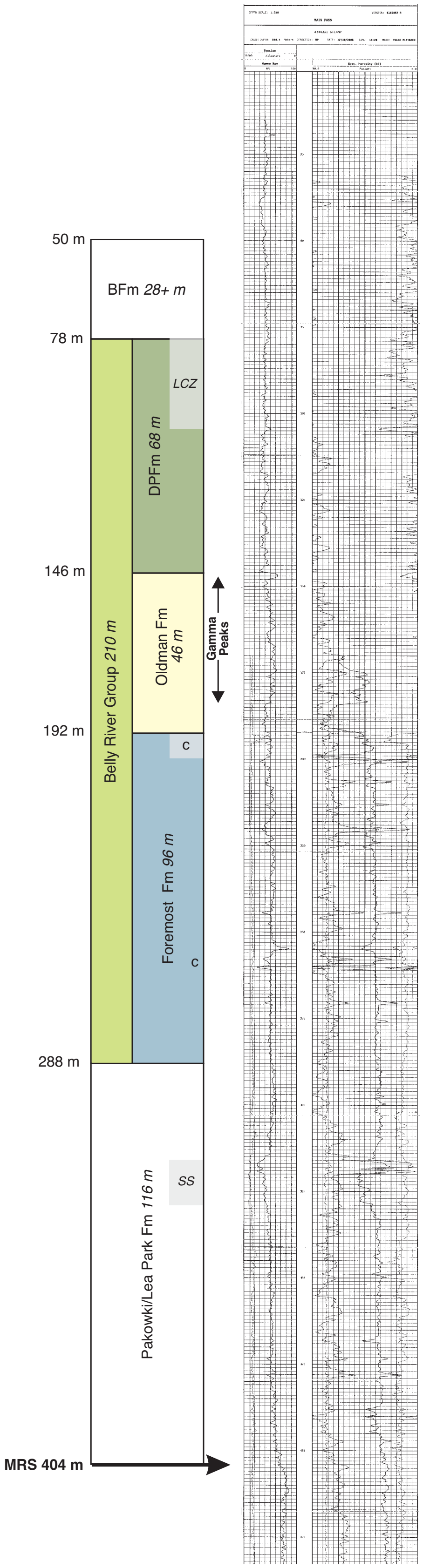

#28
15-30-28-02W4

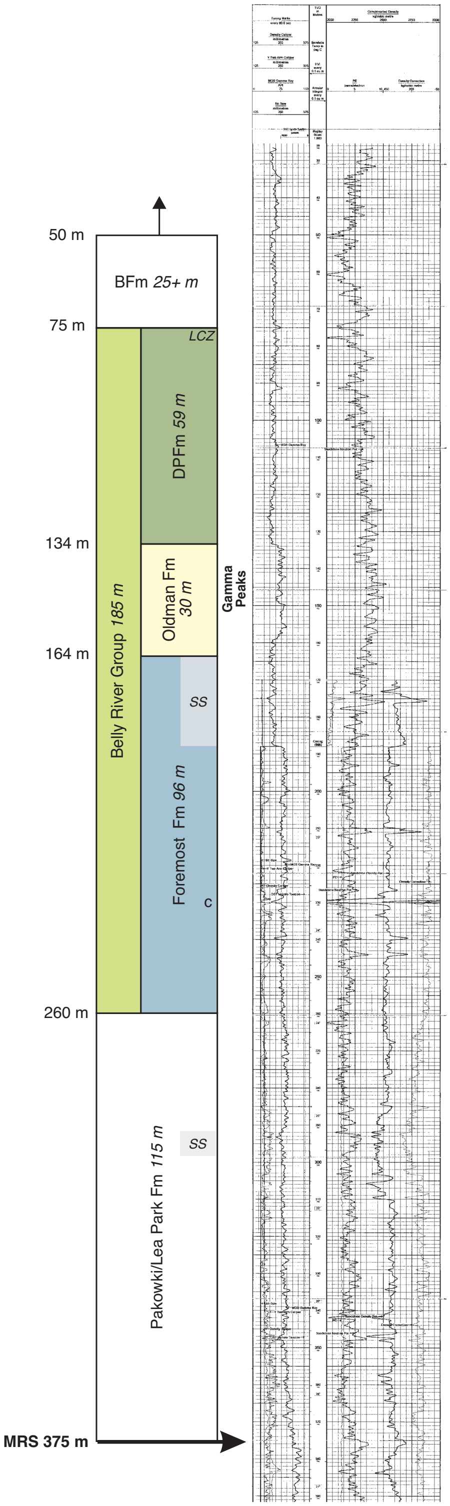

#29
08-28-29-03W4

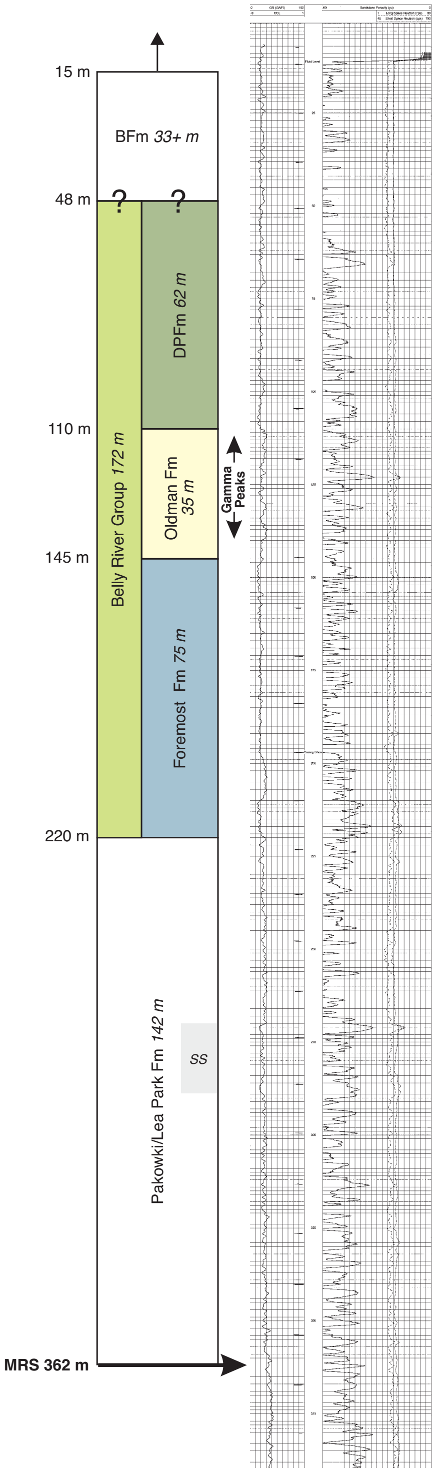

#30
16-36-30-01W4

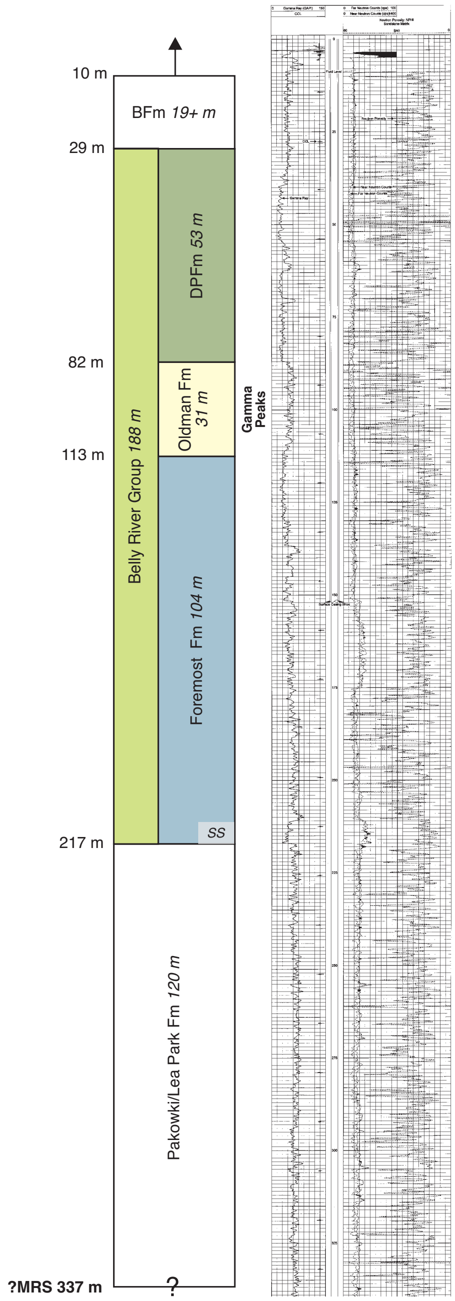

#31
07-07-33-01W4

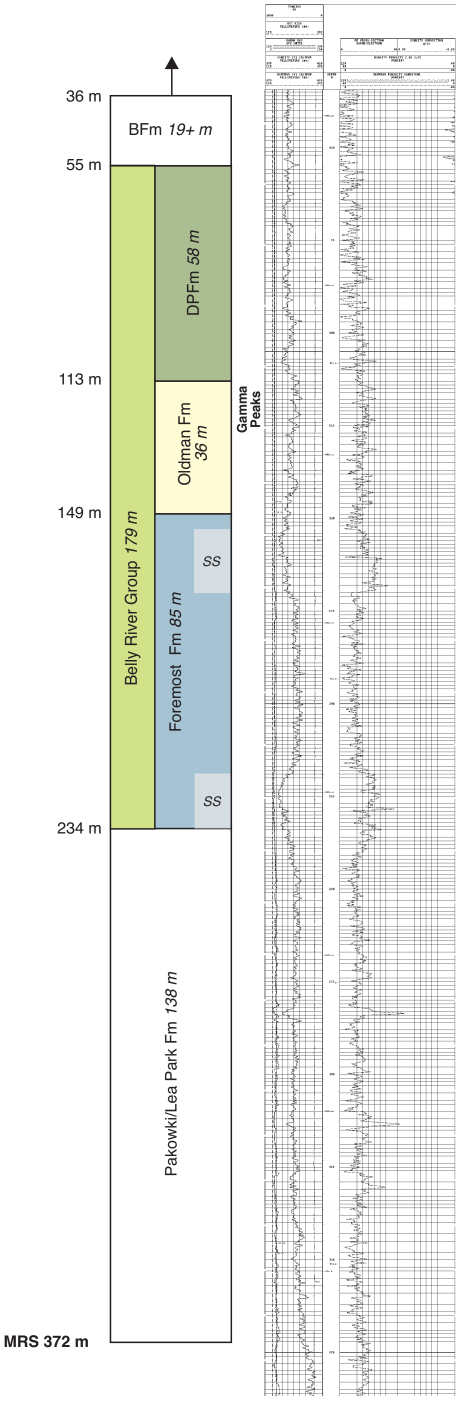

#32
Reference well
13-13-34-01W4

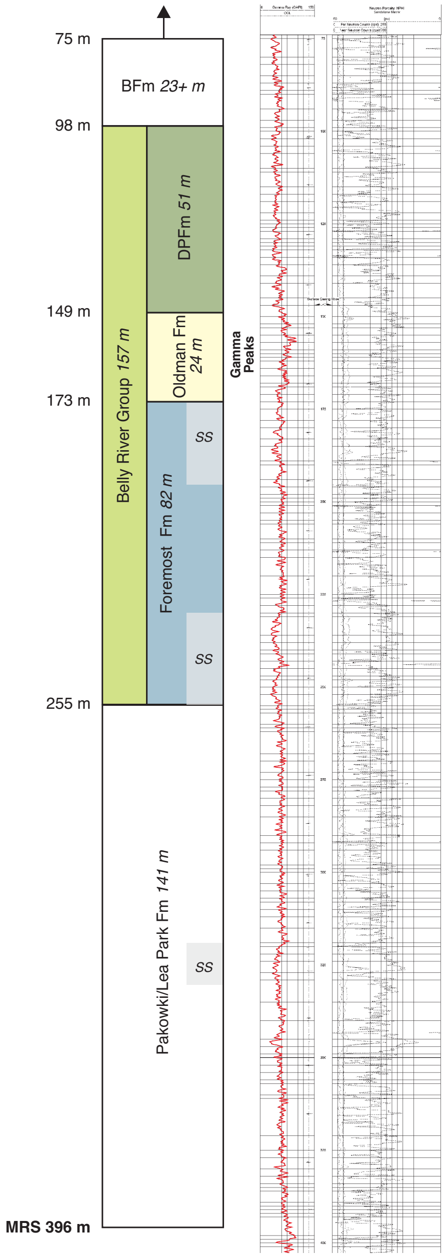

#33
16-01-36-02W4

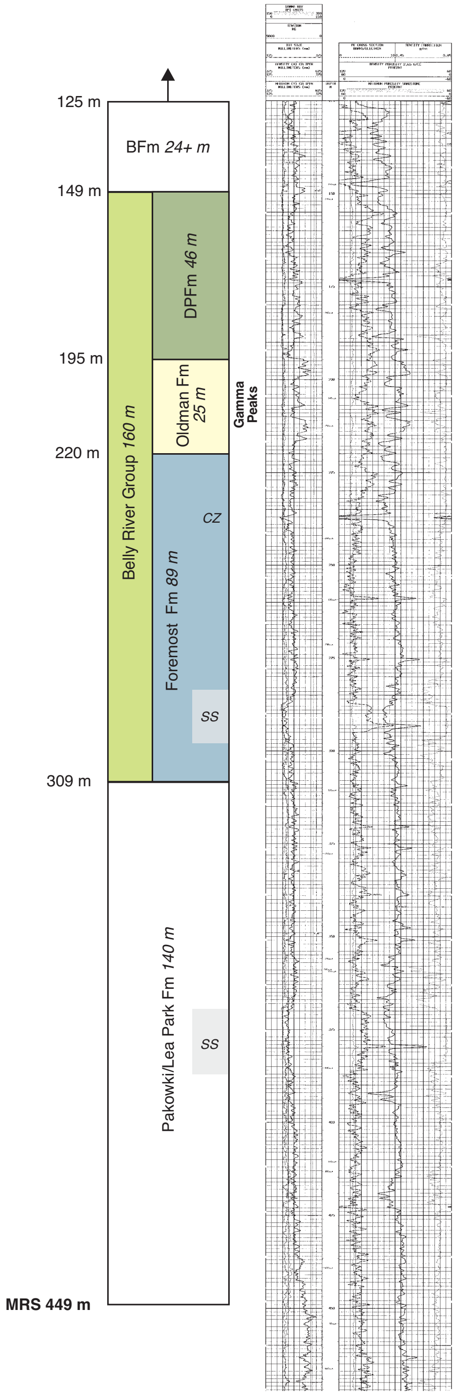

#34
Reference well
3-27-38-02W4

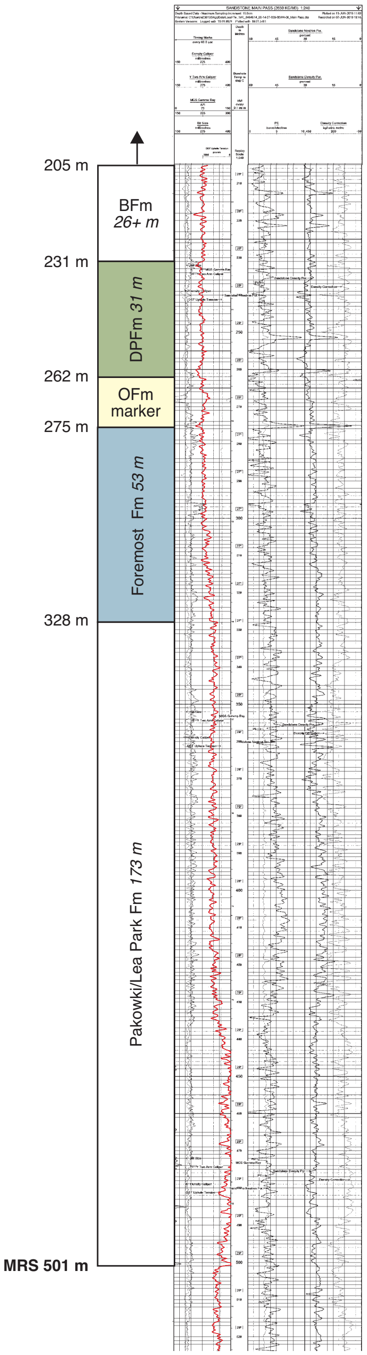

#35
10-34-40-04W4

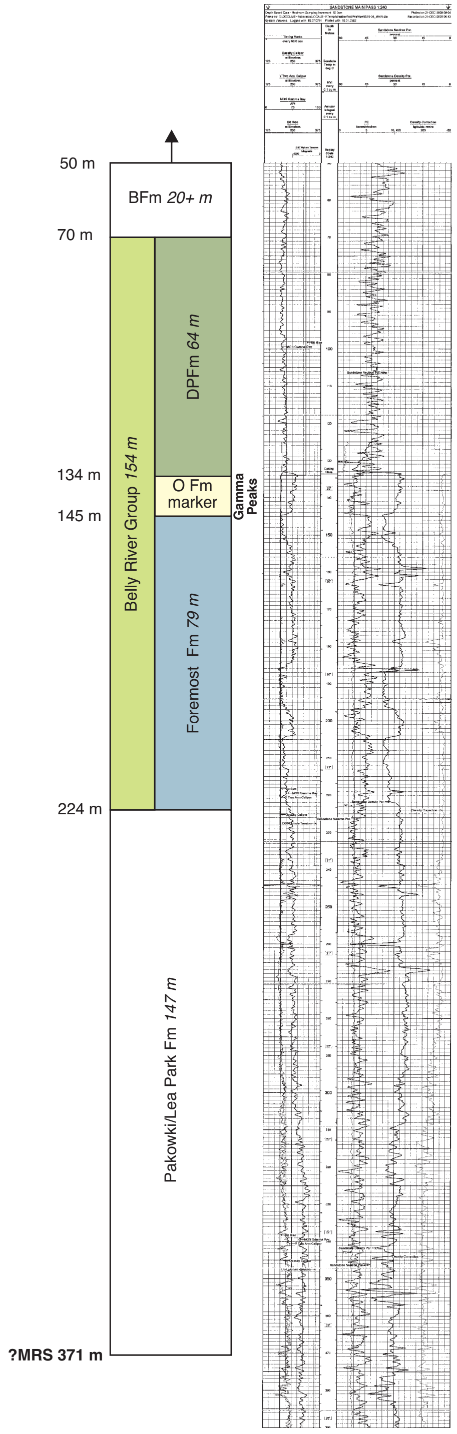

Supplement: S1 Fig — (PDF) [file pone.0292318.s001.pdf]
