# Supplementary material for: Stratigraphic architecture of the Belly River Group (Campanian, Cretaceous) in the plains of southern Alberta: Revisions and updates to an existing model and implications for correlating dinosaur-rich strata
Source: PLoS One. 2024 Jan 25;19(1):e0292318. doi: 10.1371/journal.pone.0292318 (PMC10810474; doi:10.1371/journal.pone.0292318)

#21
Reference well
06-12-21-01W4

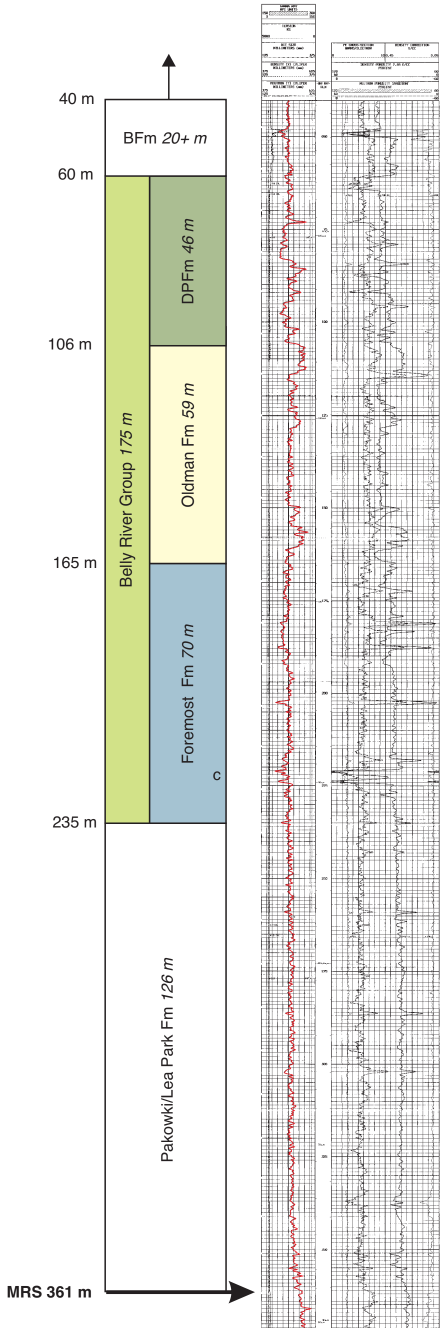

#36
Reference well
07-28-21-02W4

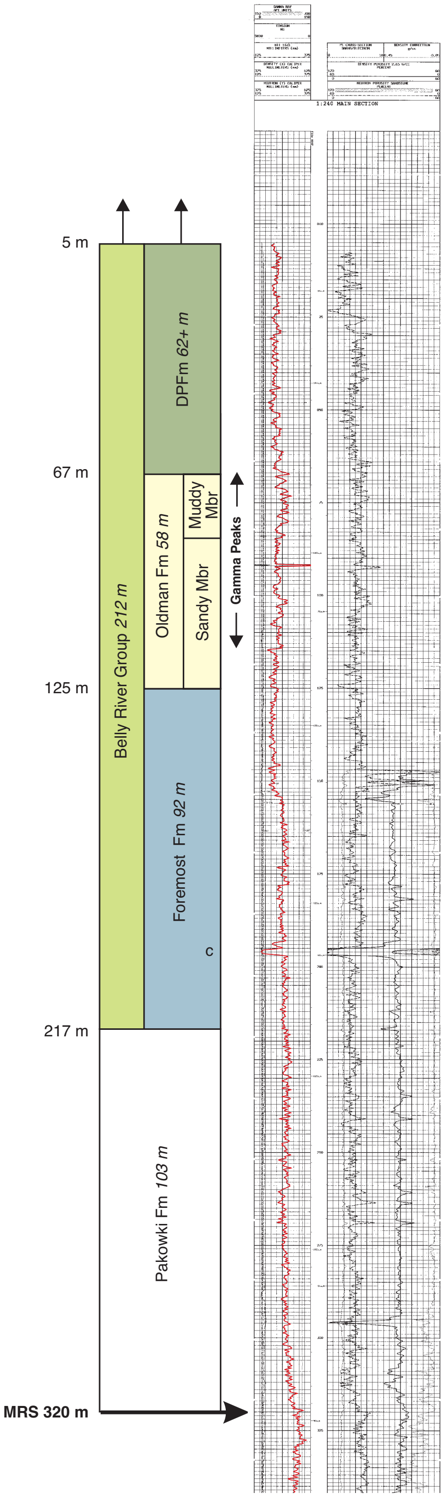

#37
14-12-21-03W4

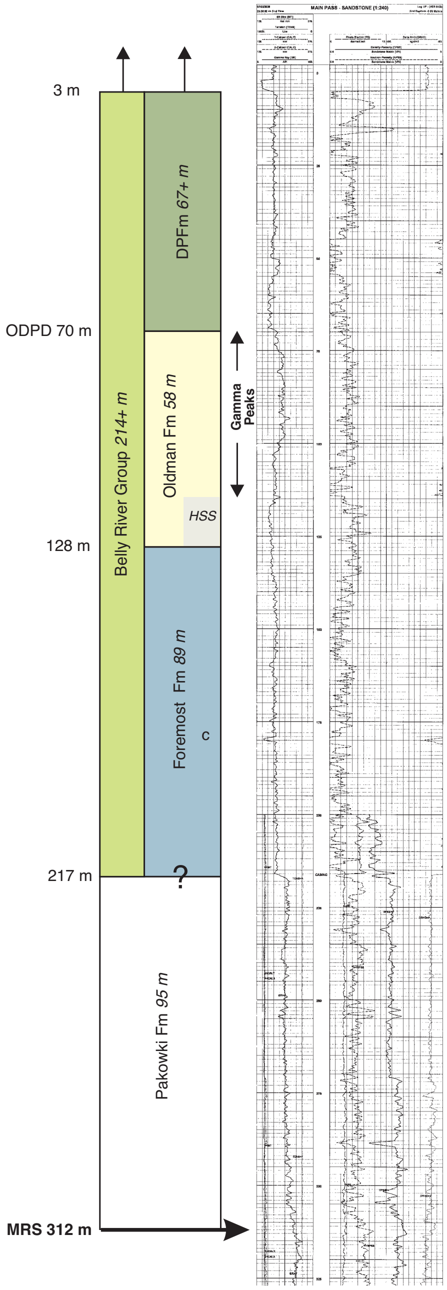

#38
08-08-21-04W4

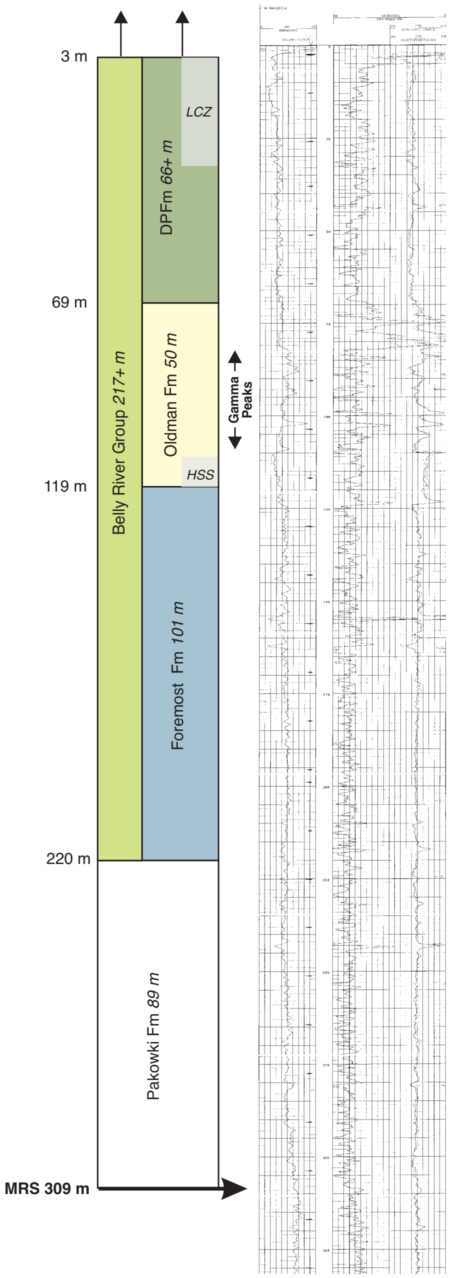

#39
14-01-21-05W4

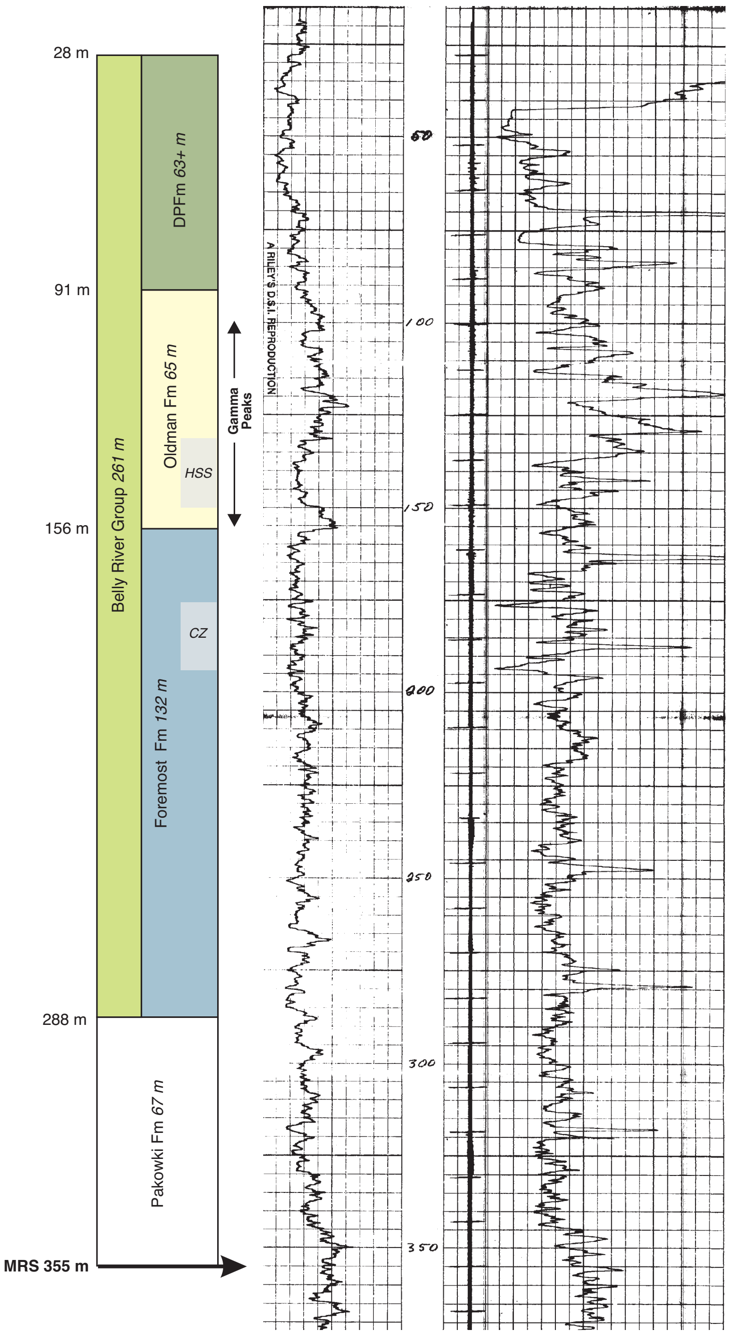

#40
06-13-21-06W4

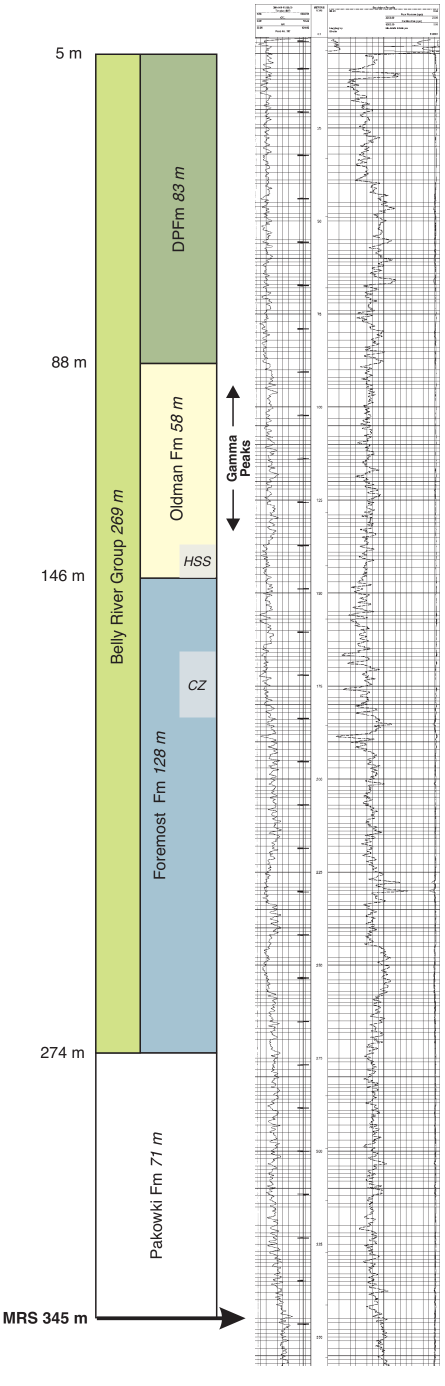

#41
15-10-21-07W4

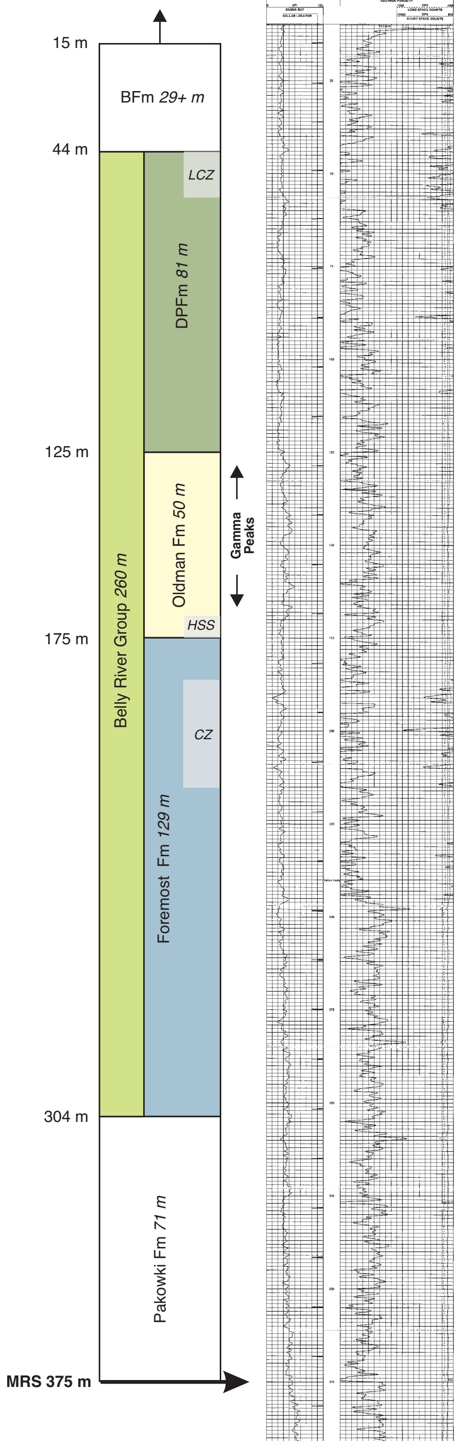

#42
02-05-21-08W4

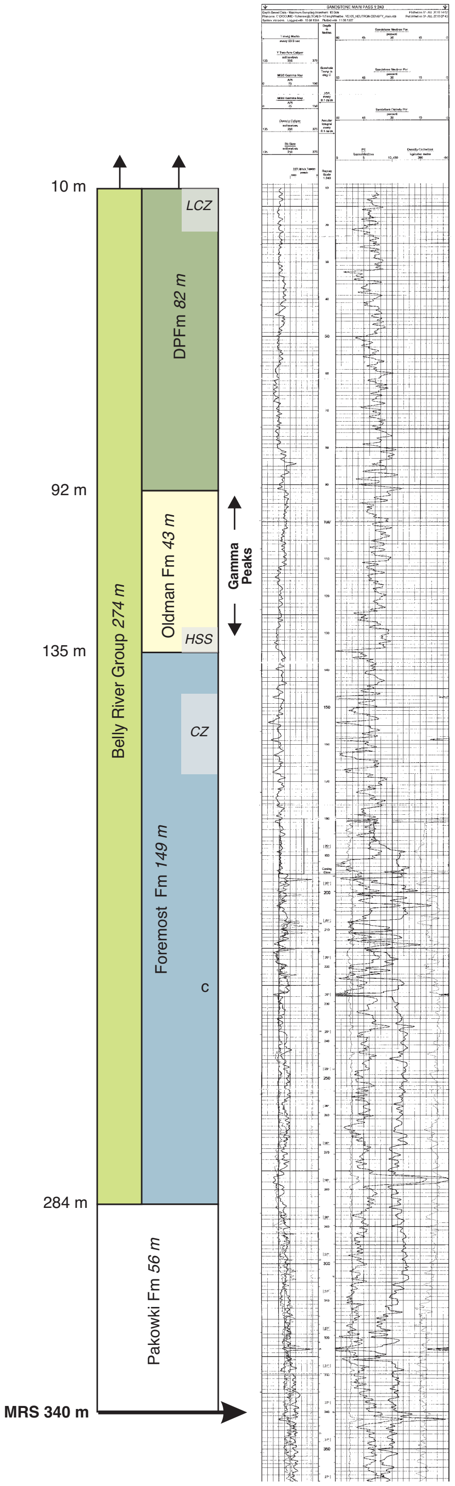

#43
14-04-21-09W4

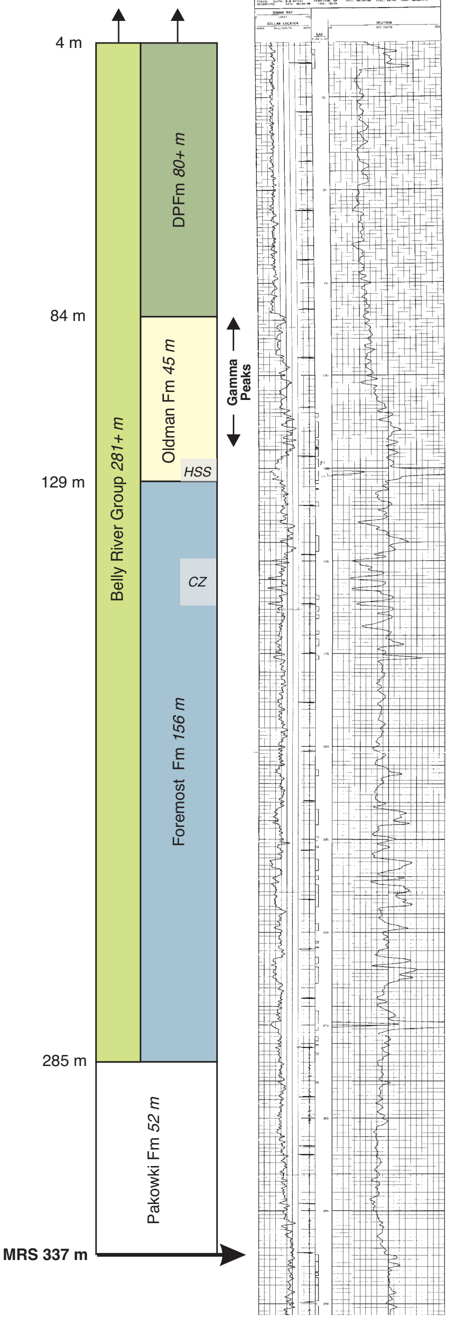

#44
Reference well
04-02-21-10W4

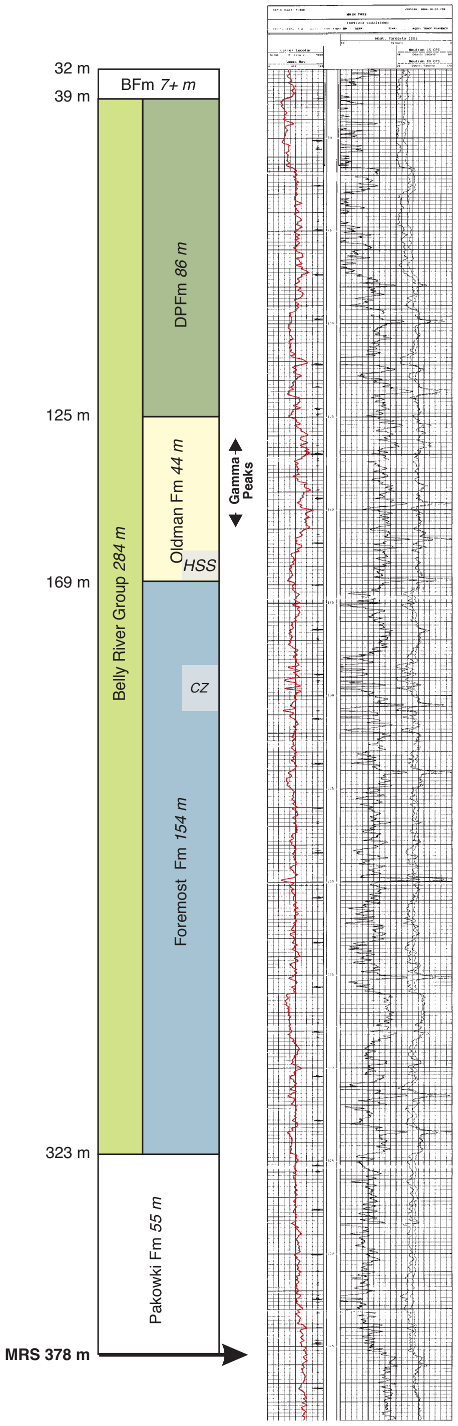

#45
05-05-20-11W4

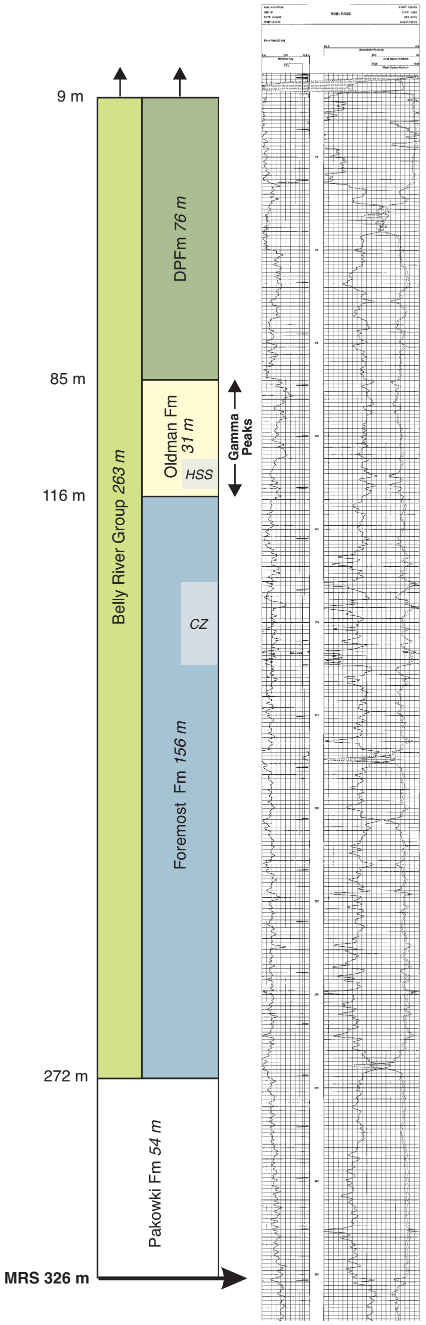

#46
08-17-20-12W4

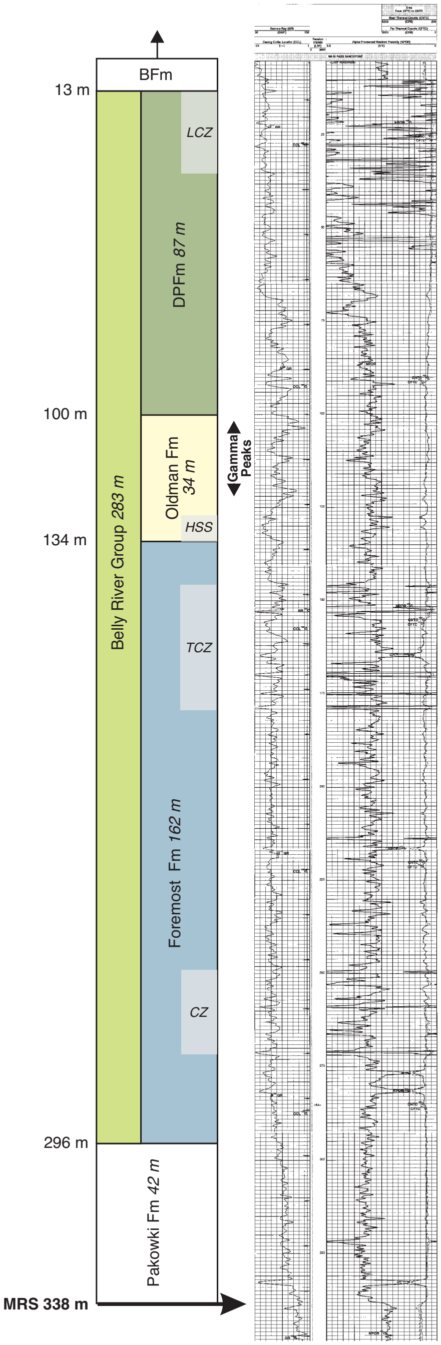

#47
01-34-21-13W4

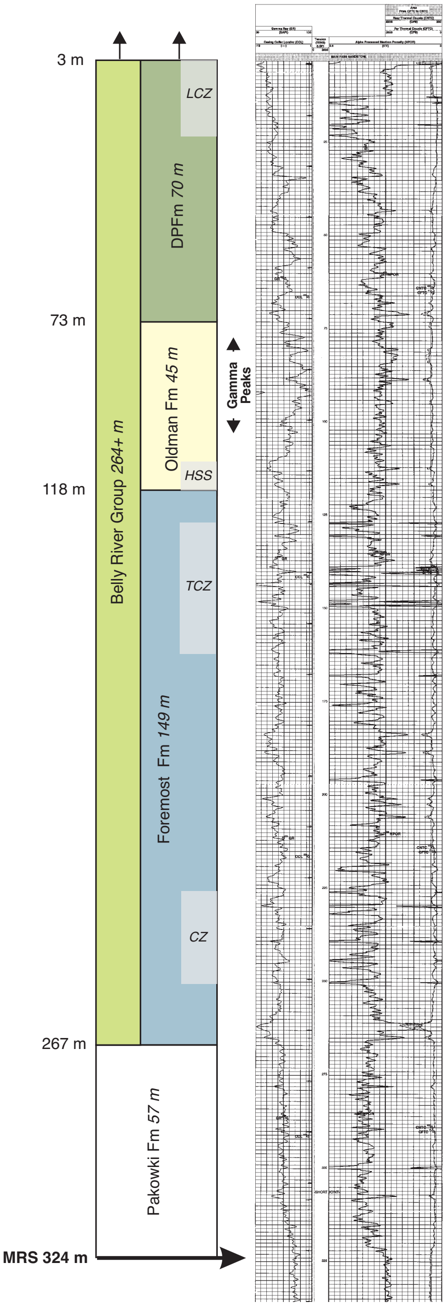

#48
05-22-21-14W4

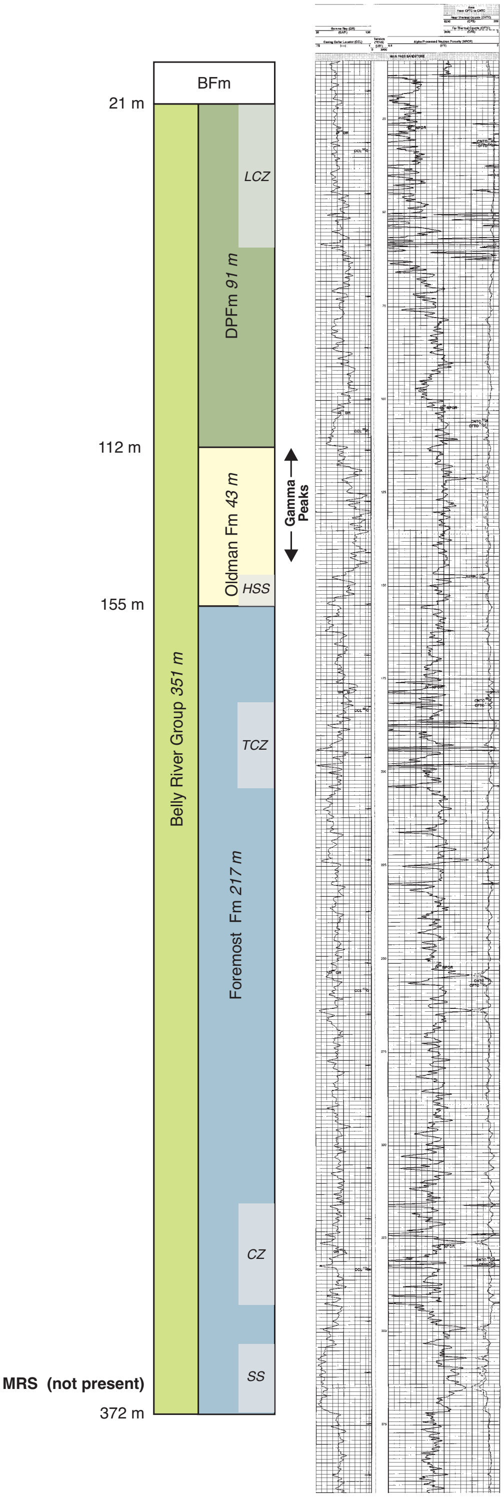

#49
15-10-21-15W4

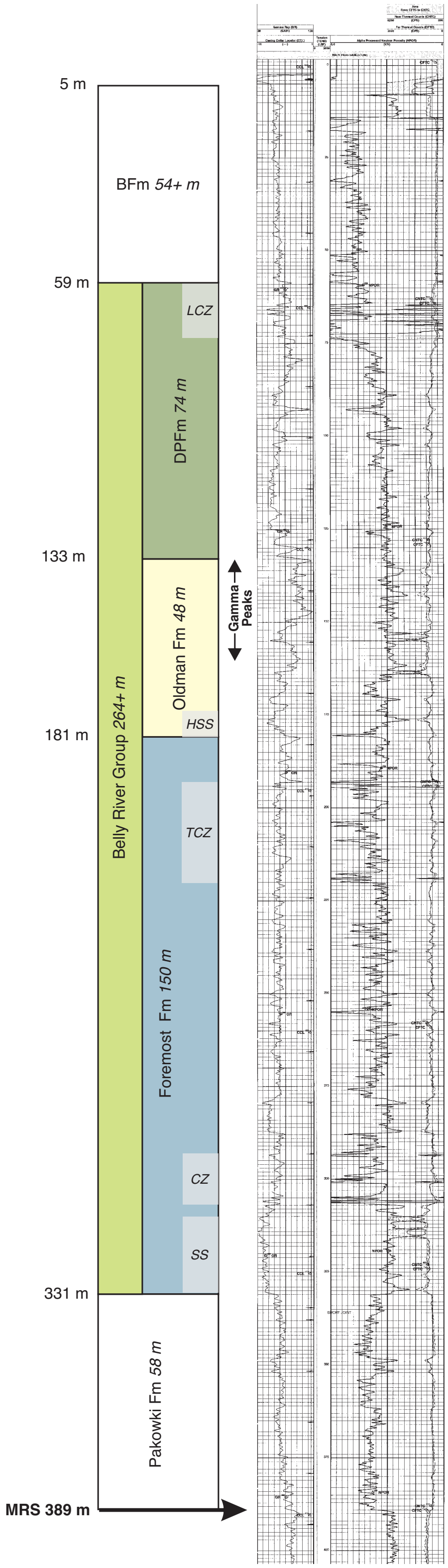

#50
10-16-21-16W4

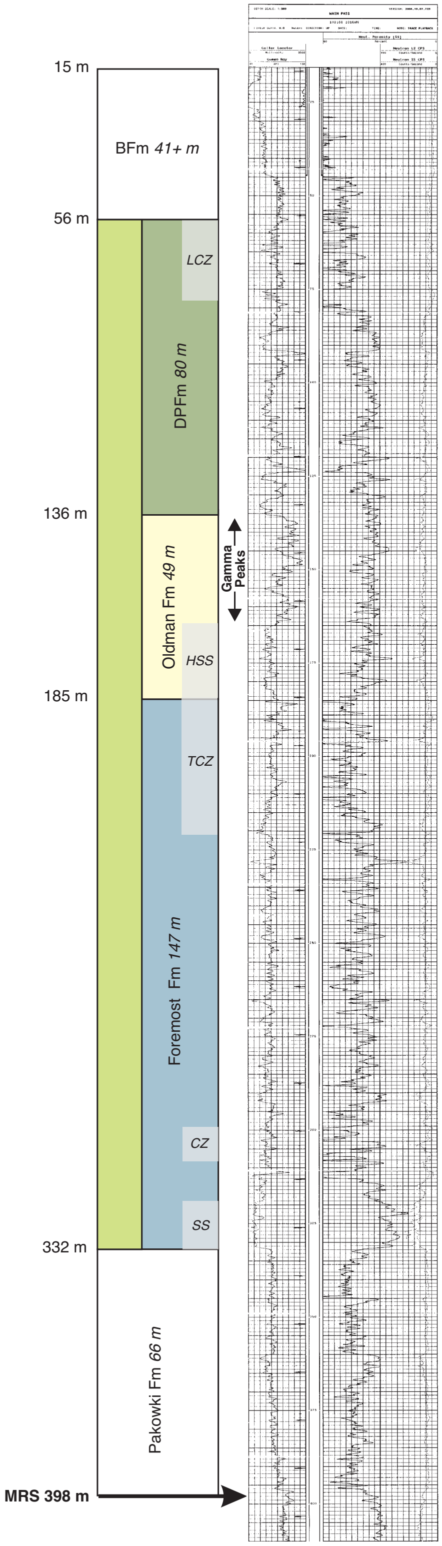

#51
02-21-21-17W4

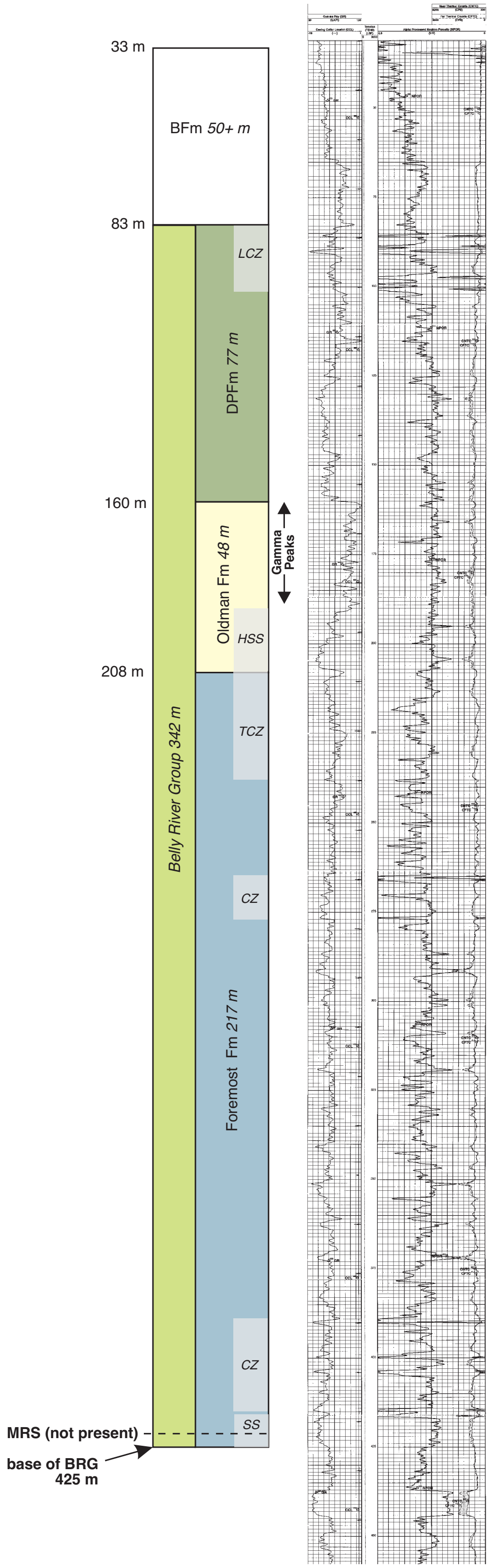

#52
08-35-21-18W4

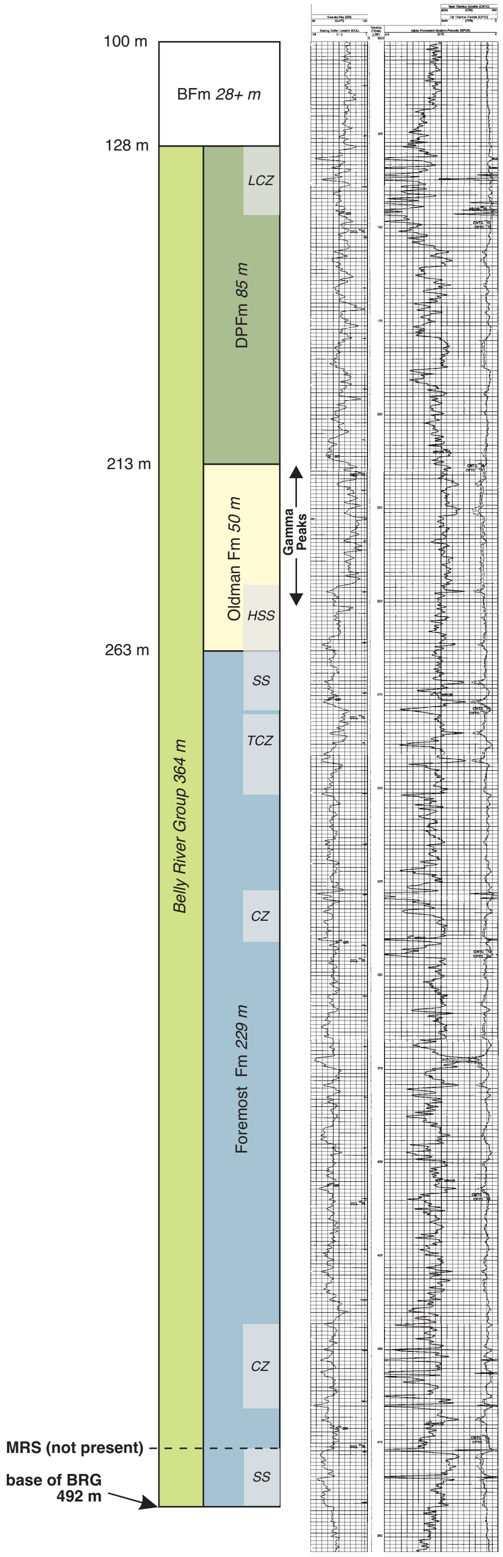

#53
Reference well
08-34-21-20W4

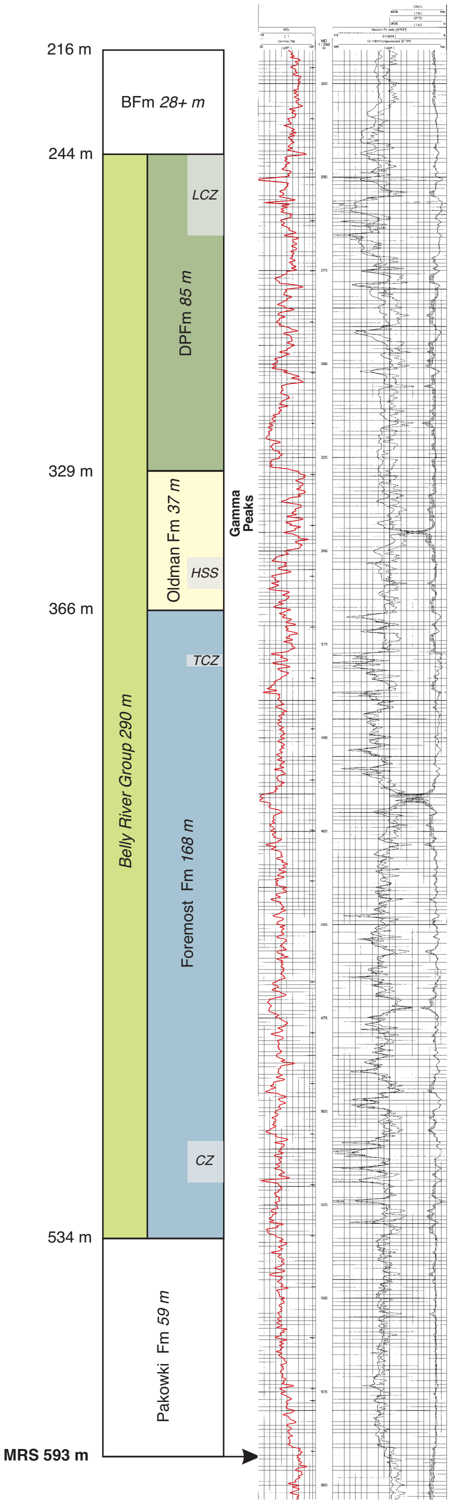

#54
11-32-21-21W4

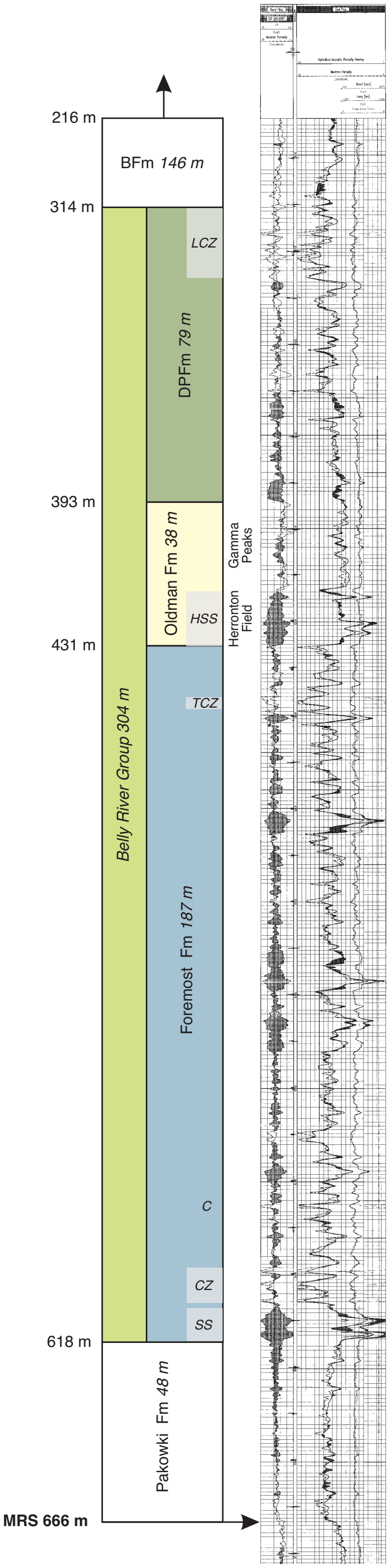

#55
14-29-21-22W4

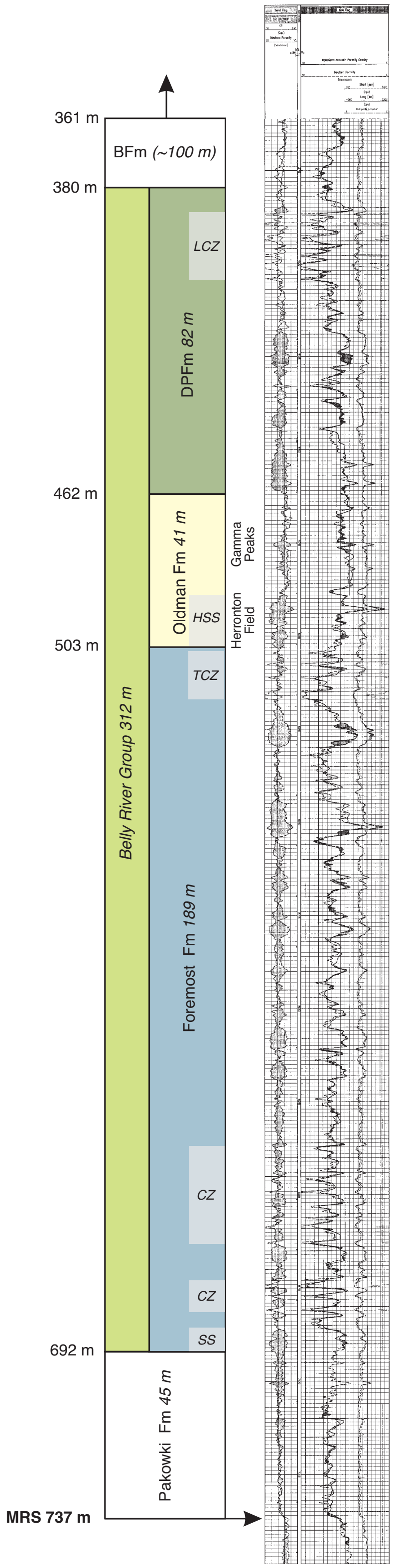

#56
14-30-21-23W4

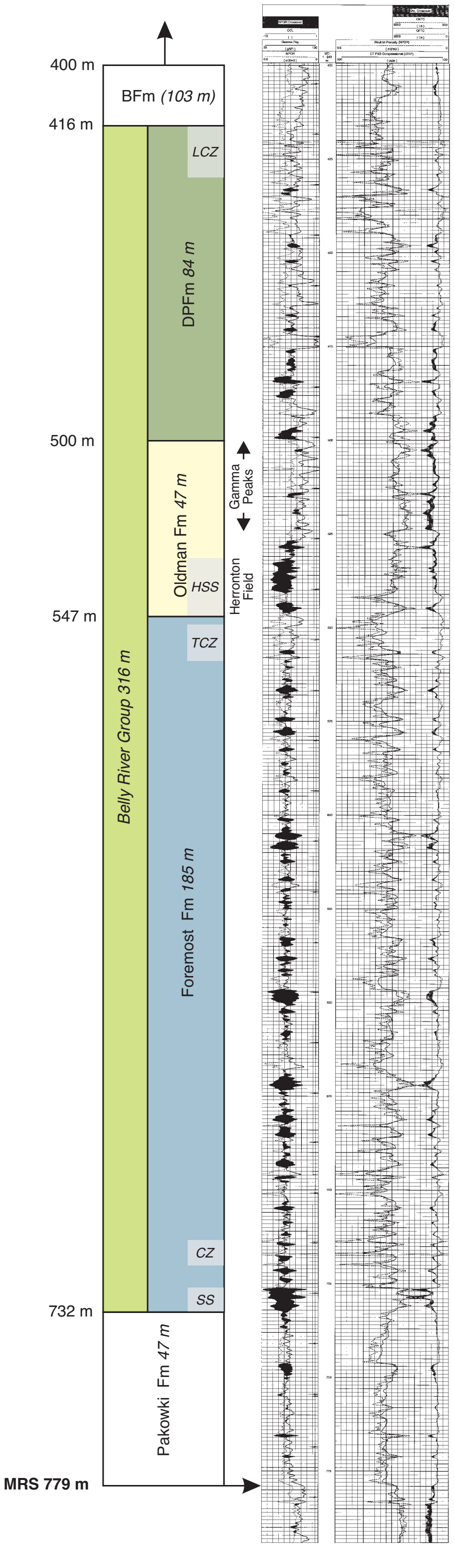

#57
14-35-21-24W4

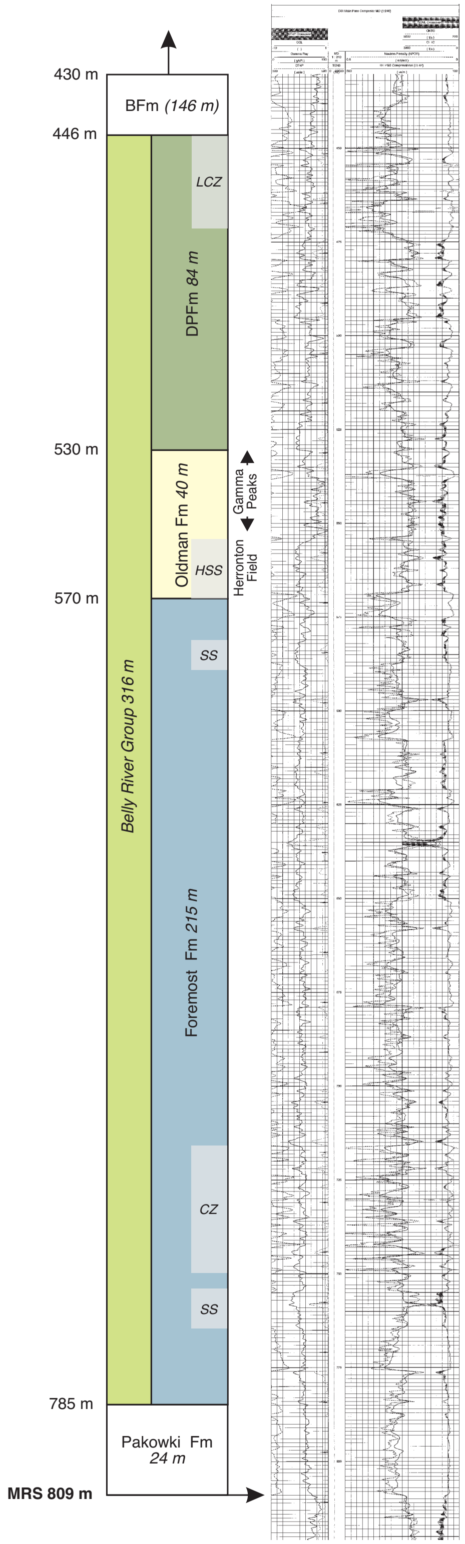

#58
03-15-21-25W4

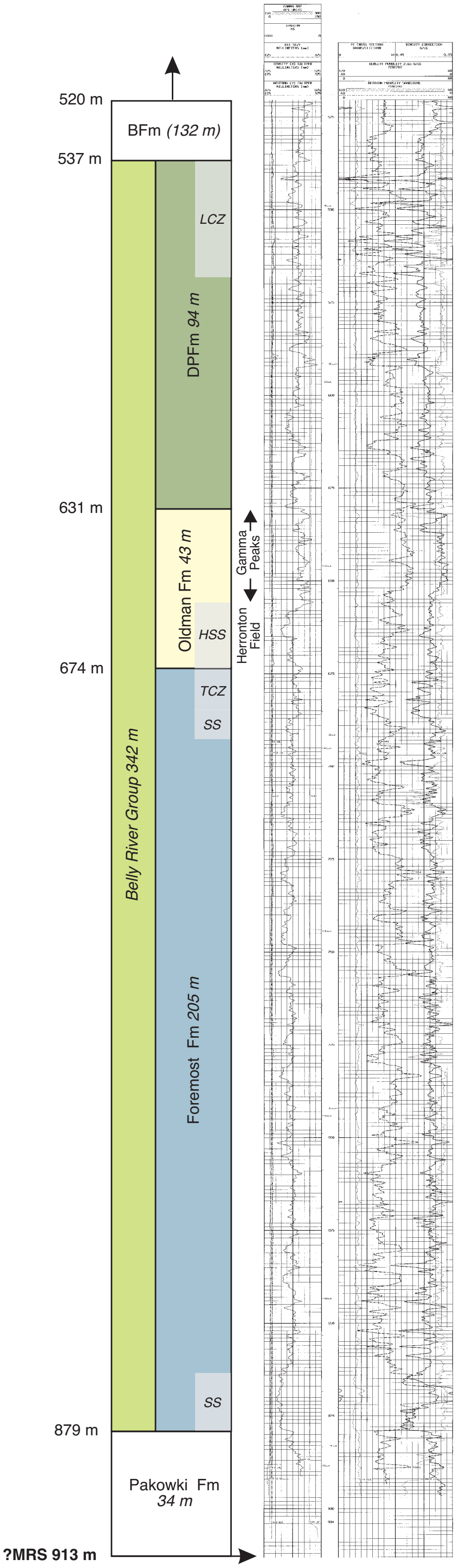

#59
06-22-21-26W4

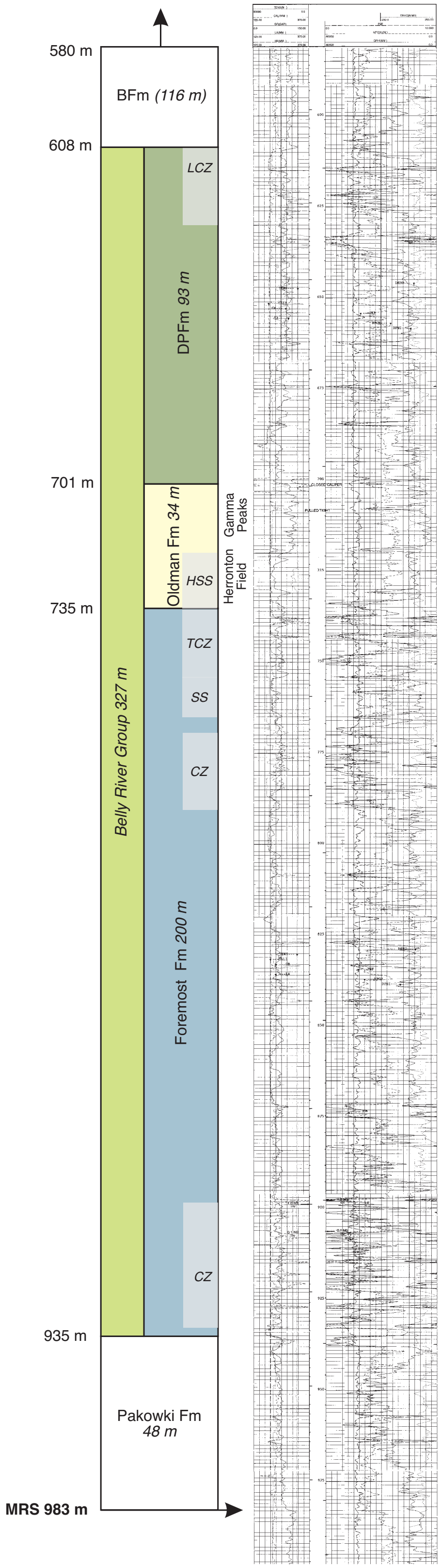

#60
11-02-21-27W4

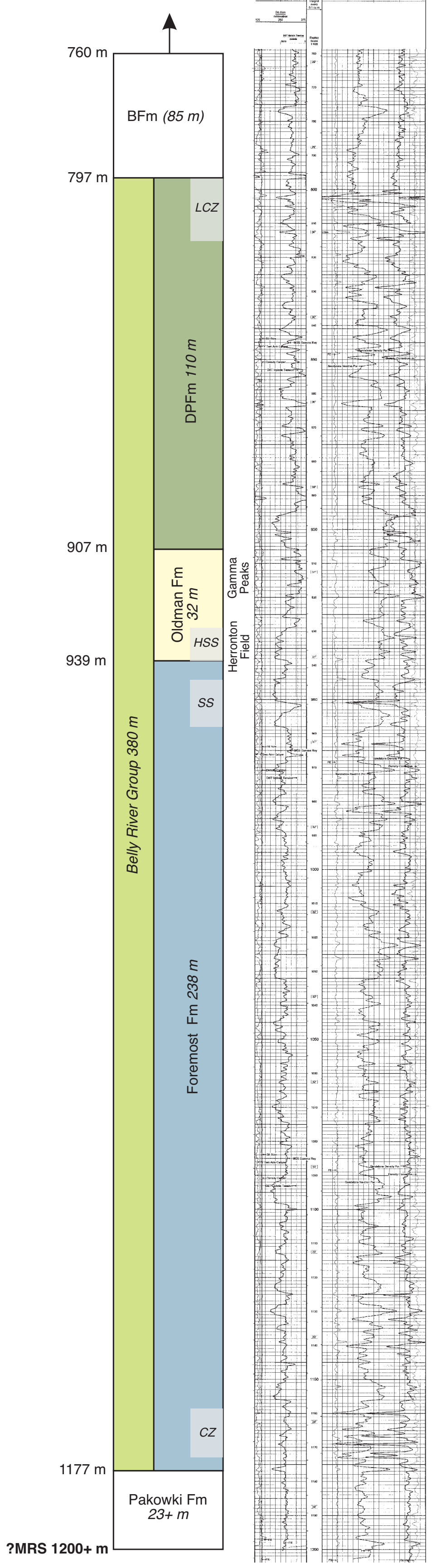

#61
03-20-21-28W4

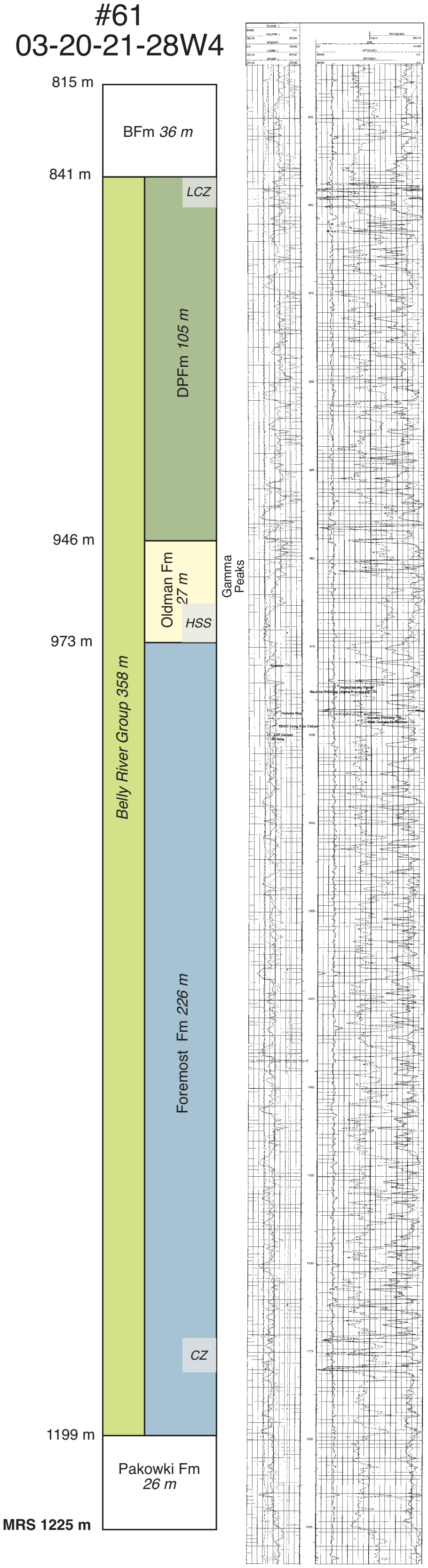

#62
11-36-21-29W4

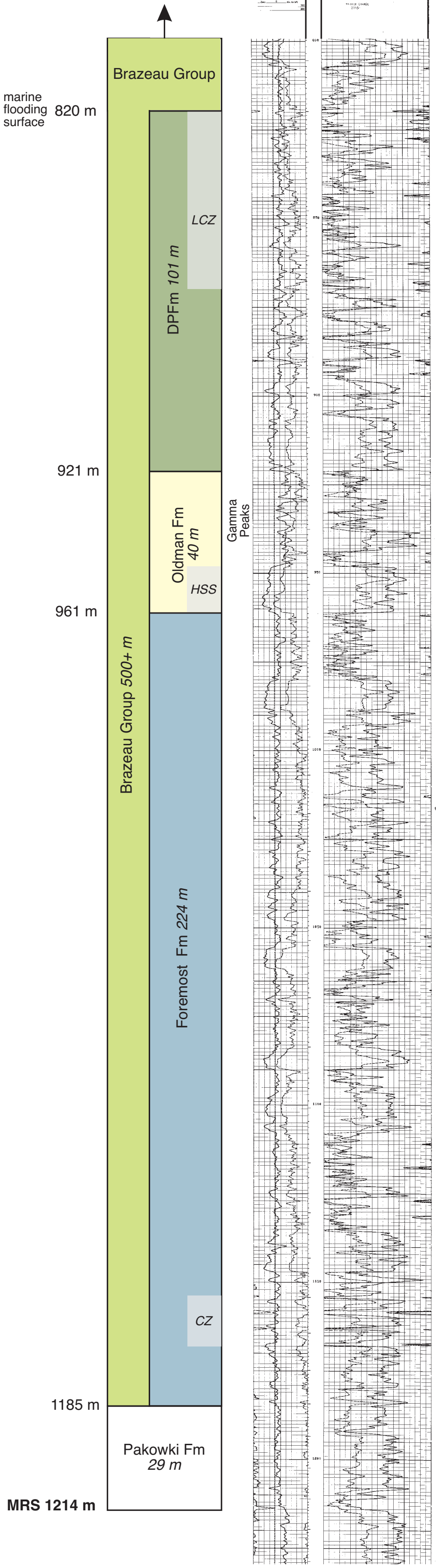

Supplement: S2 Fig — (PDF) [file pone.0292318.s002.pdf]
