# Supplementary material for: Stratigraphic architecture of the Belly River Group (Campanian, Cretaceous) in the plains of southern Alberta: Revisions and updates to an existing model and implications for correlating dinosaur-rich strata
Source: PLoS One. 2024 Jan 25;19(1):e0292318. doi: 10.1371/journal.pone.0292318 (PMC10810474; doi:10.1371/journal.pone.0292318)

#63 12-18-01-23W4

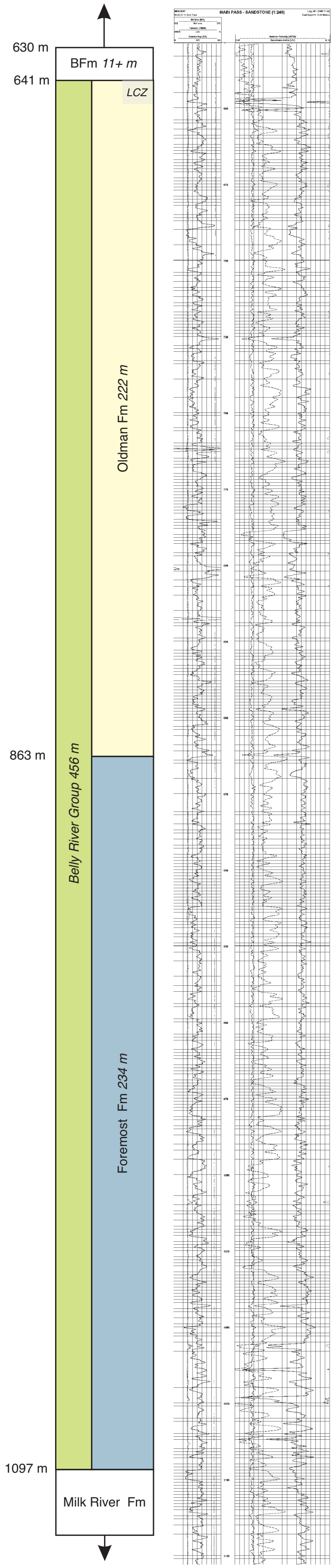

#64 01-13-02-23W4

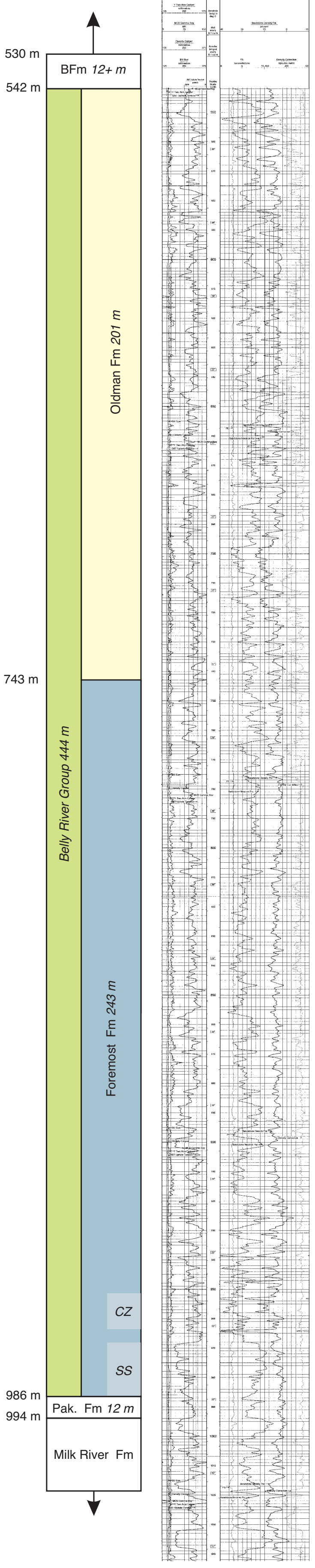

#65 Reference well
01-11-03-23W4

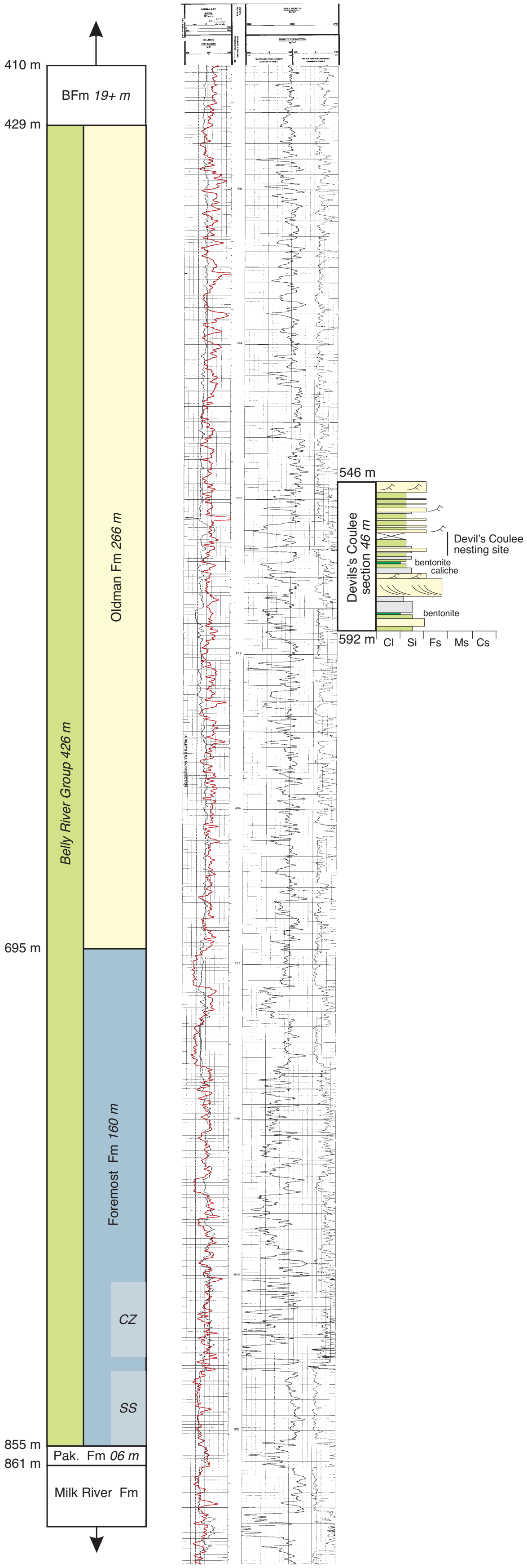

#66 03-32-04-23W4

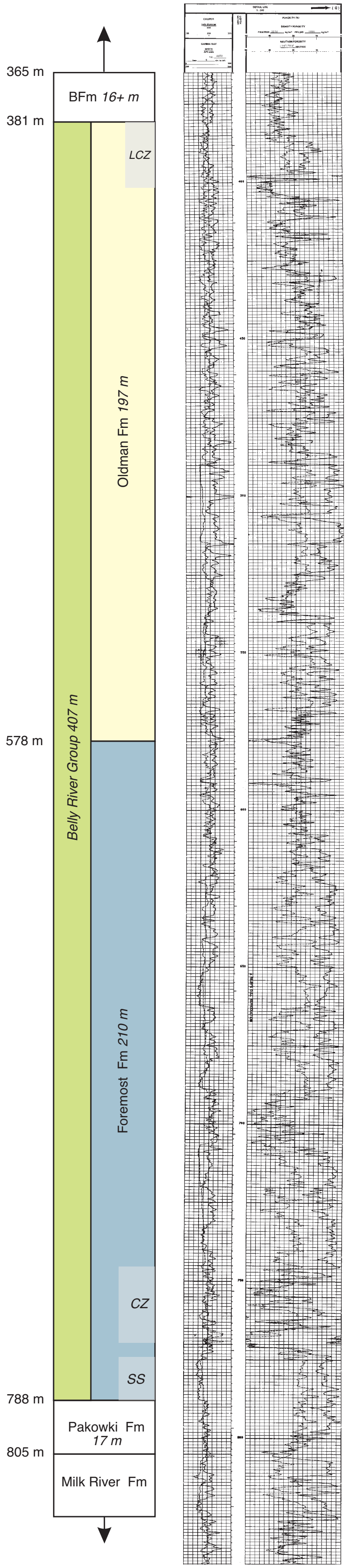

#67 09-17-05-25W4

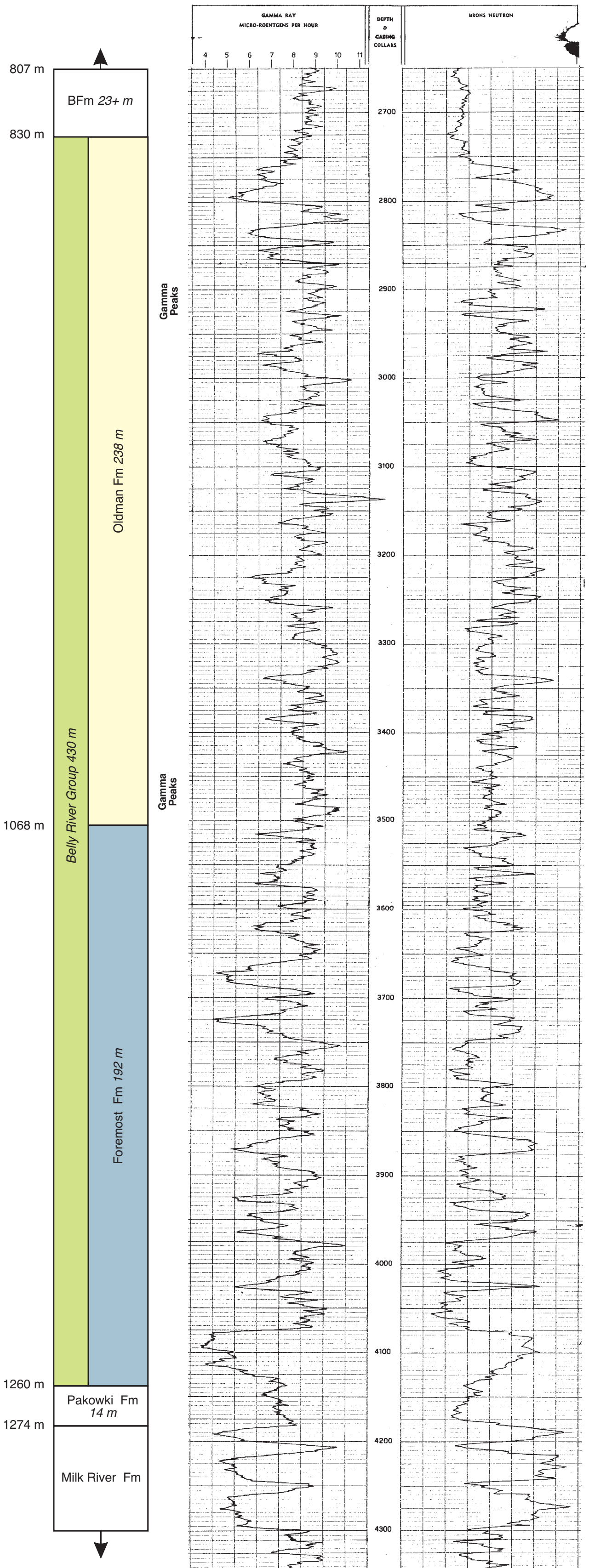

#68 Reference well
16-18-06-26W4

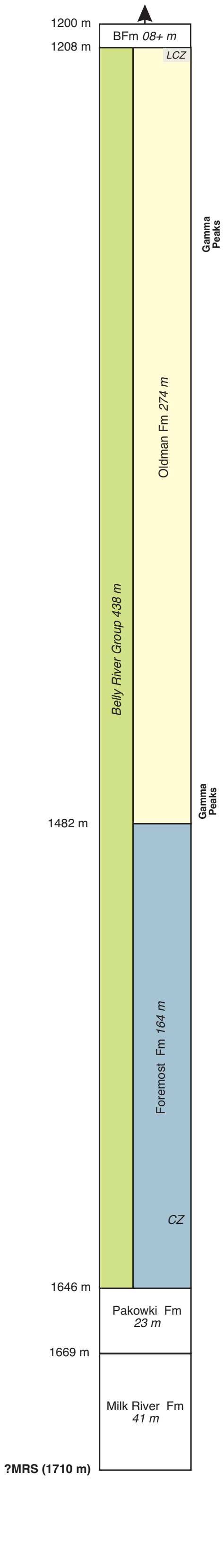

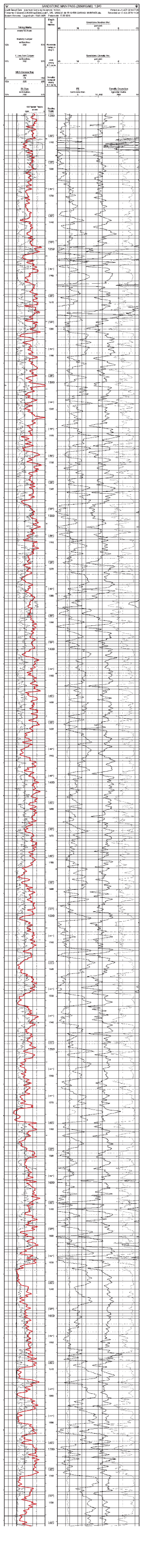

#69 10-29-07-21W4

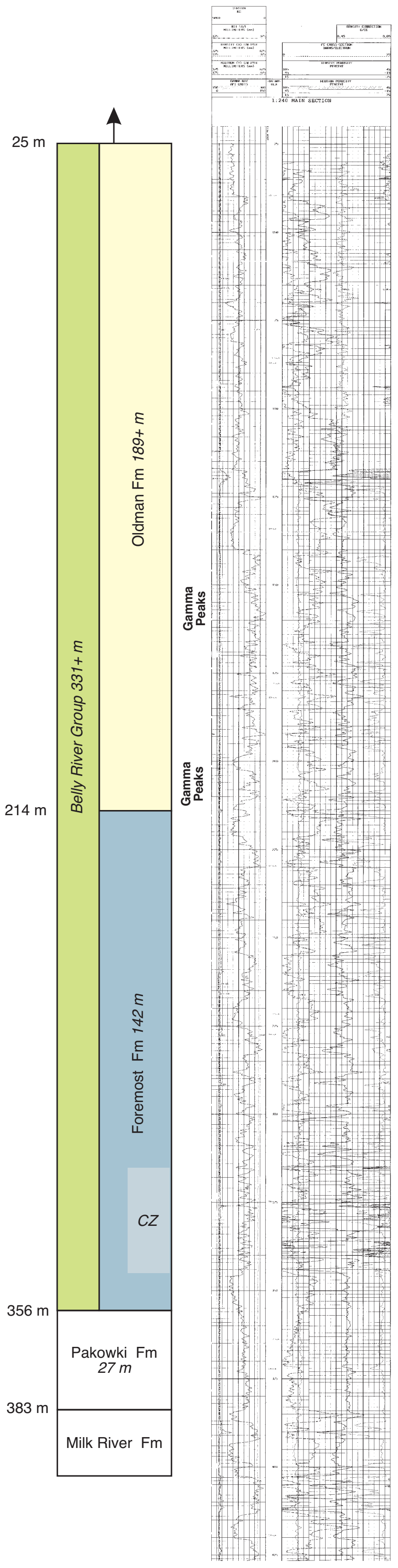

#70 15-08-08-22W4

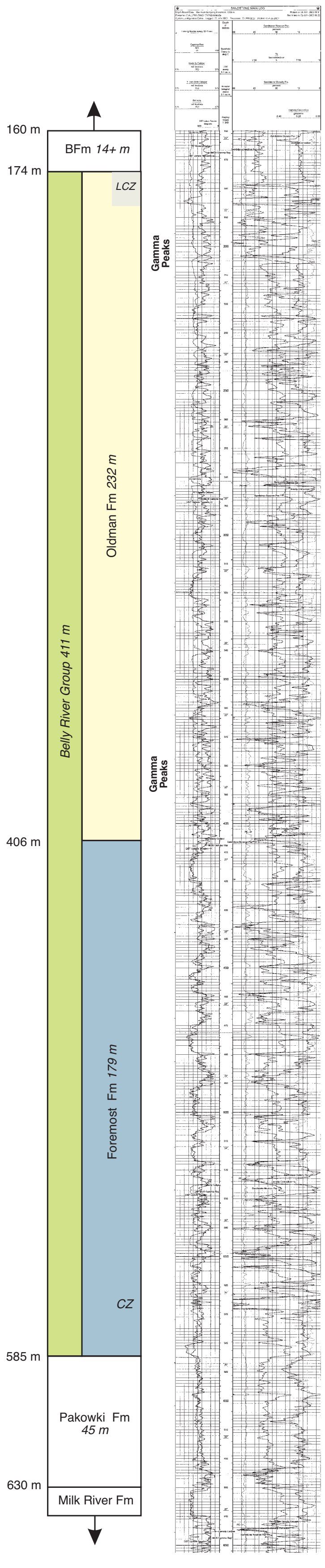

#71 02-04-09-23W4

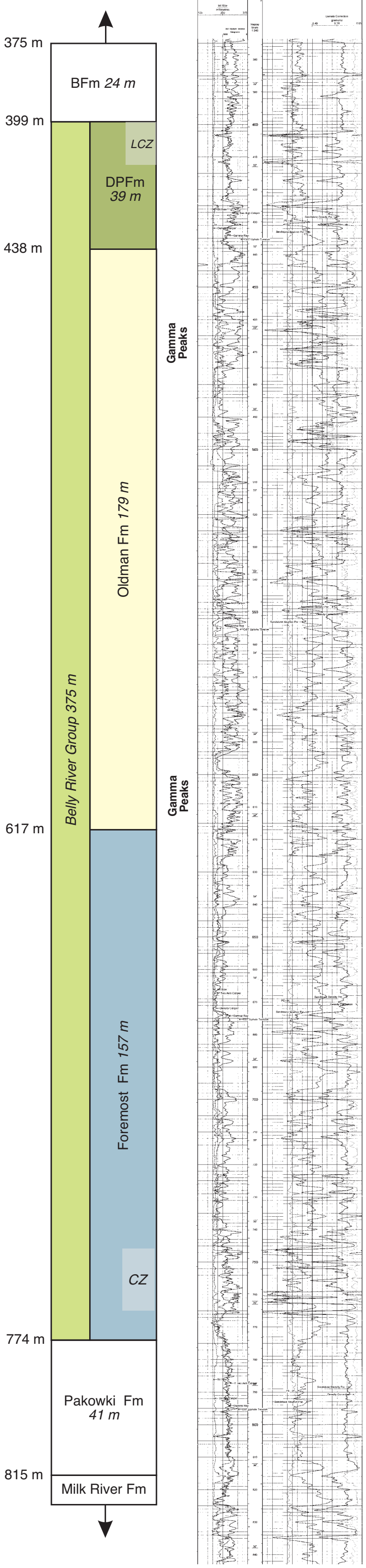

#72 13-16-10-24W4

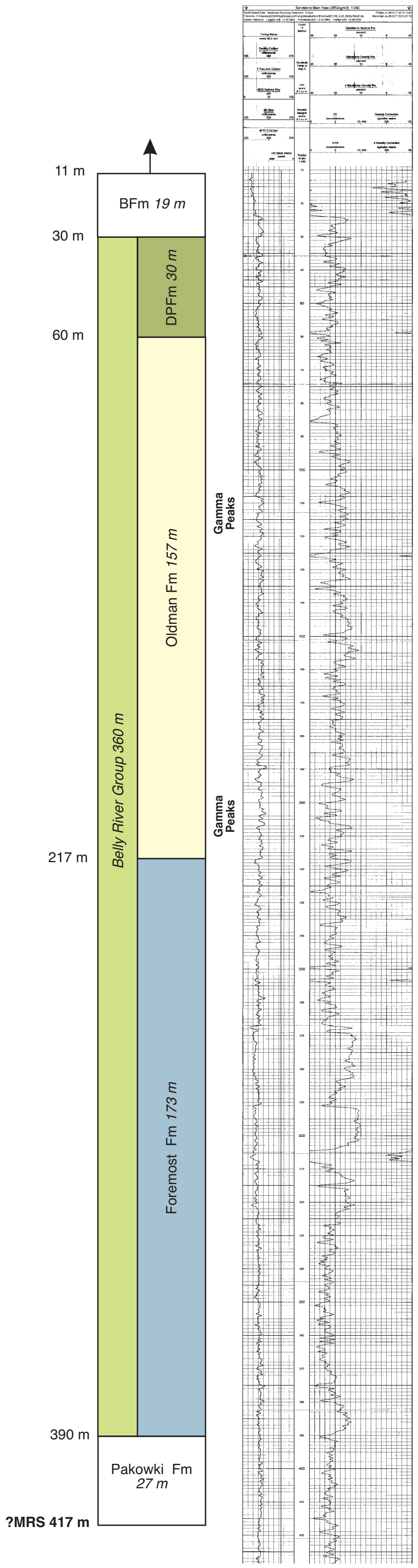

#73 05-34-11-23W4

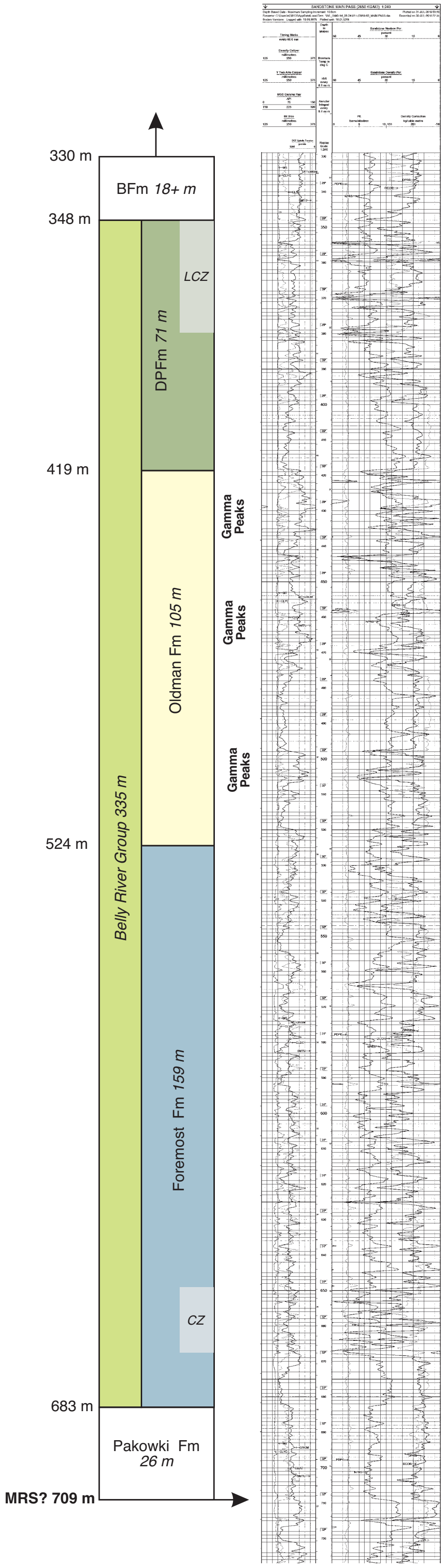

#74
03-23-12-23W4

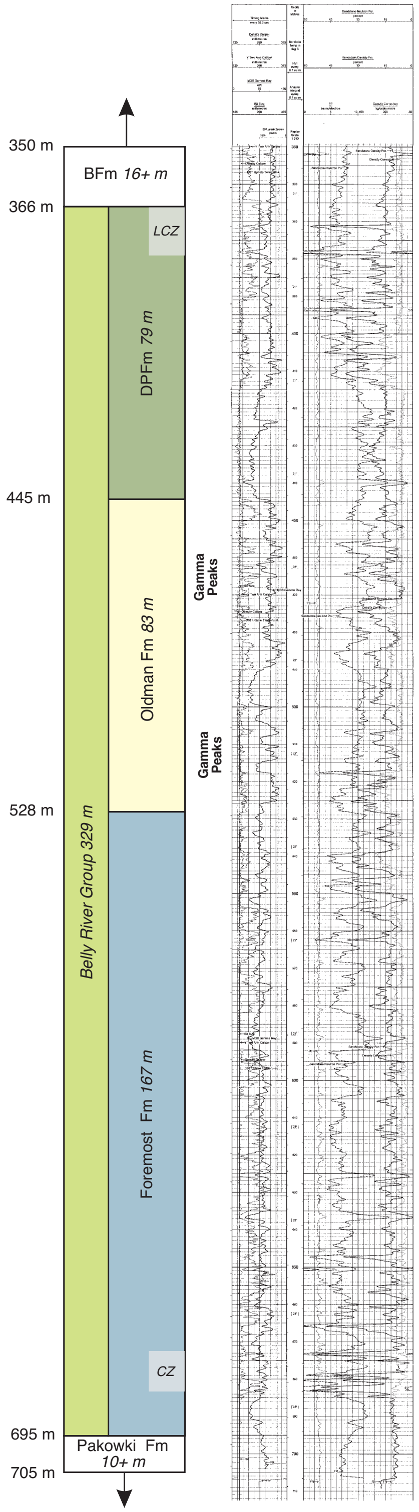

#75
04-06-13-22W4

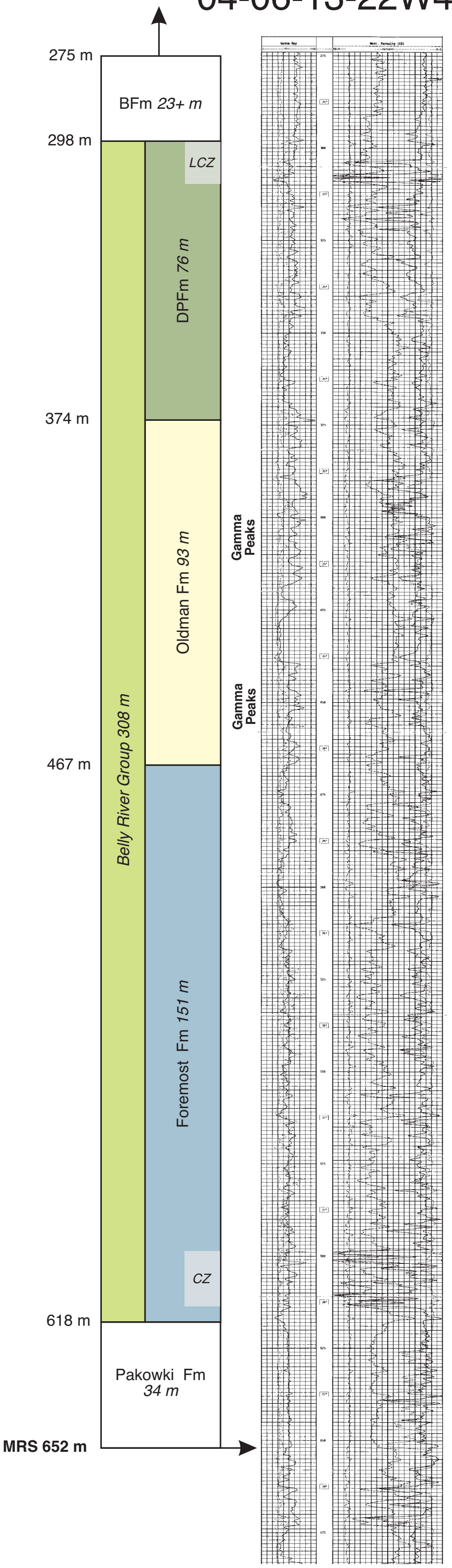

#76
06-05-14-20W4

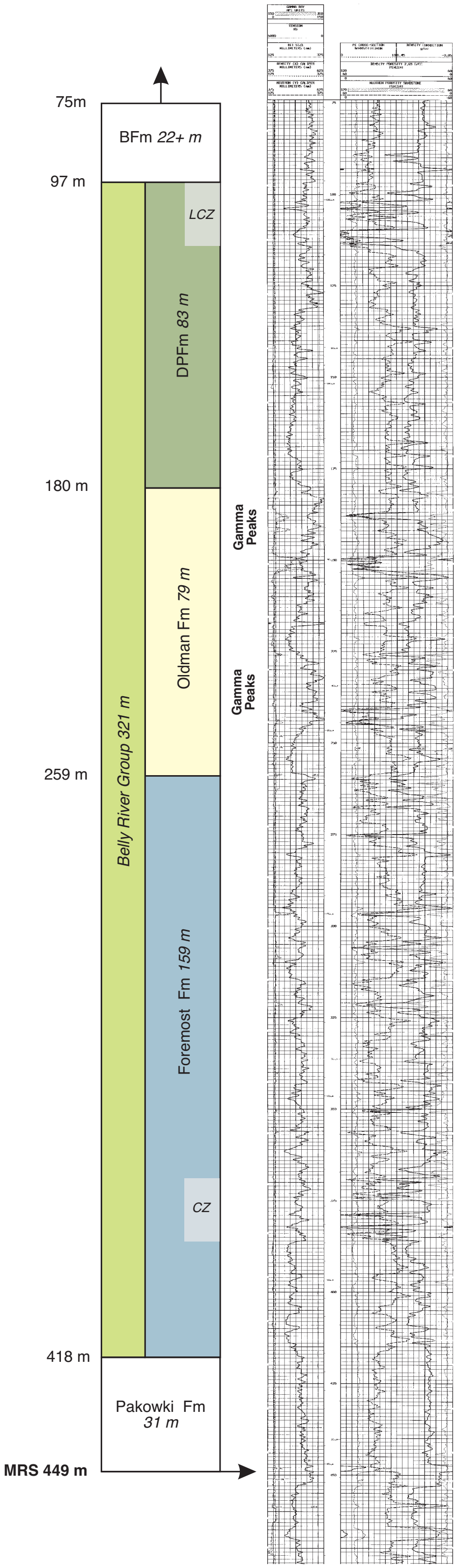

#77
01-35-15-21W4

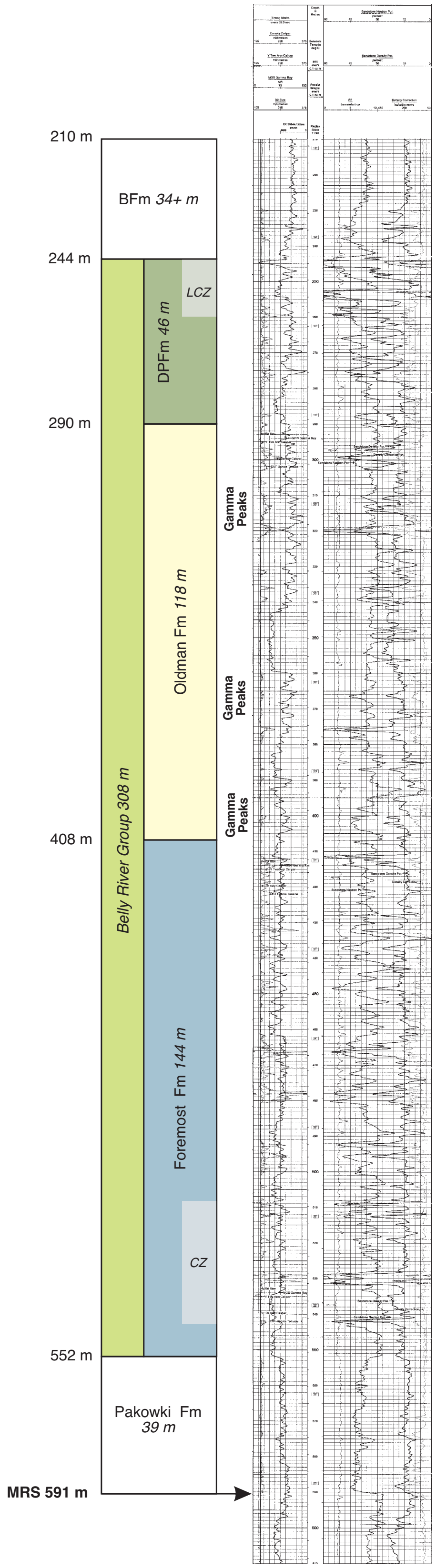

#78
14-36-16-20W4

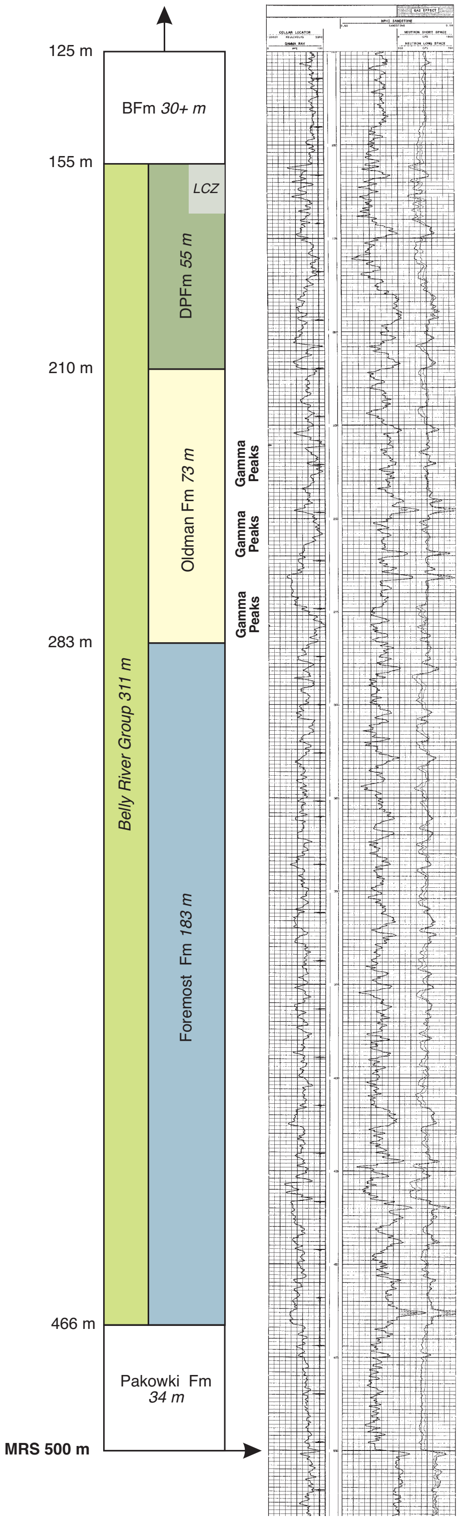

#79
06-28-17-20W4

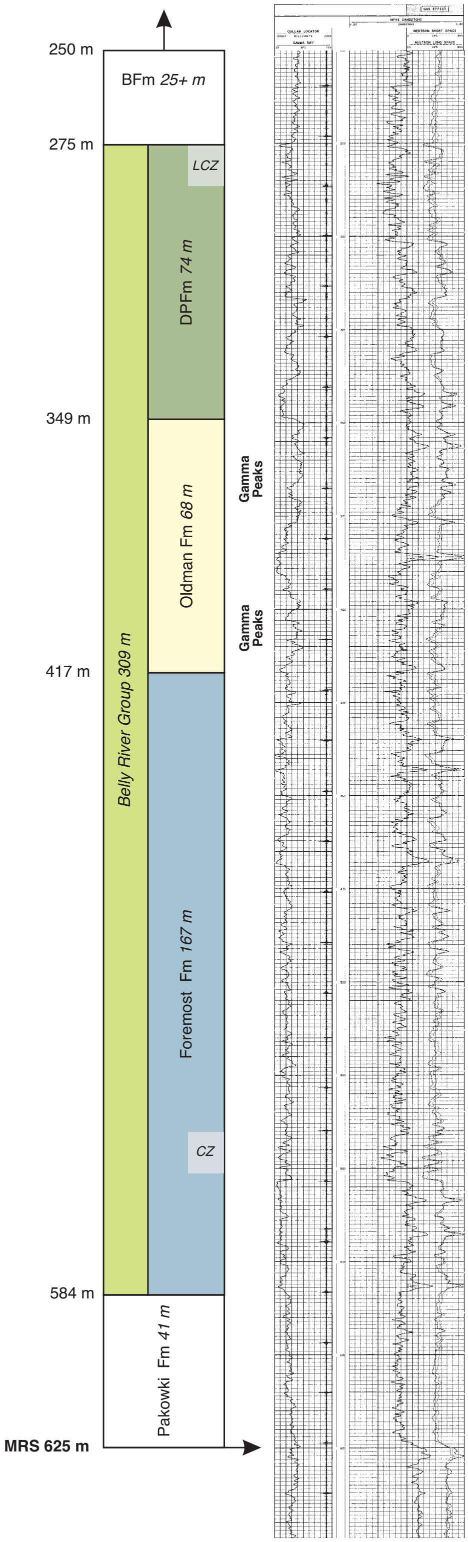

#80
06-02-18-20W4

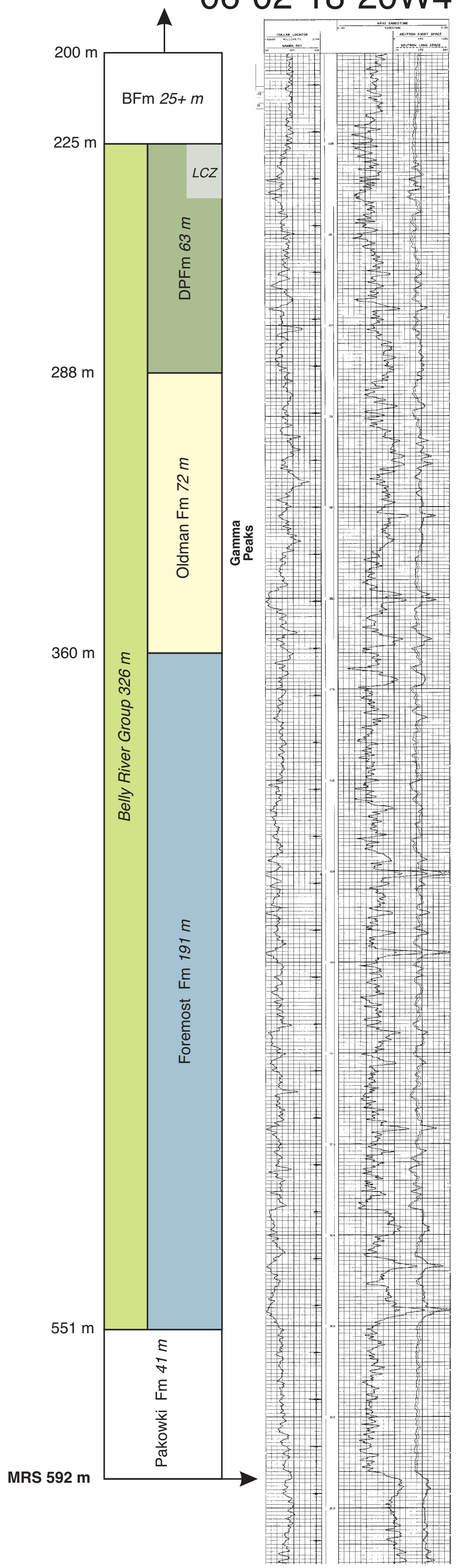

#81
14-03-19-20W4

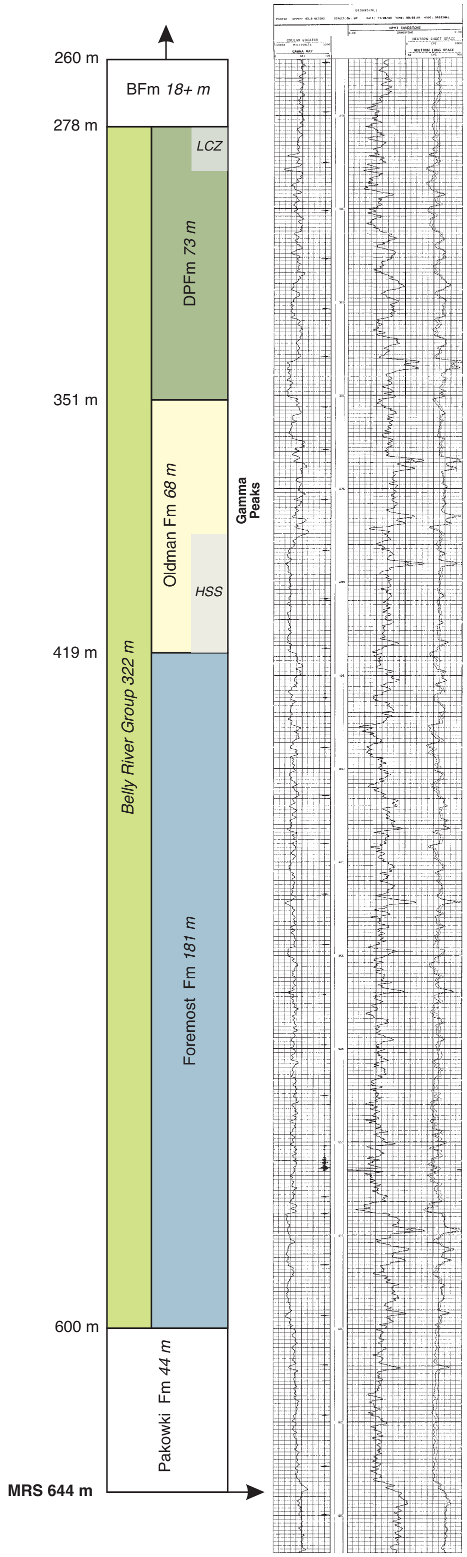

#82
04-06-20-20W4

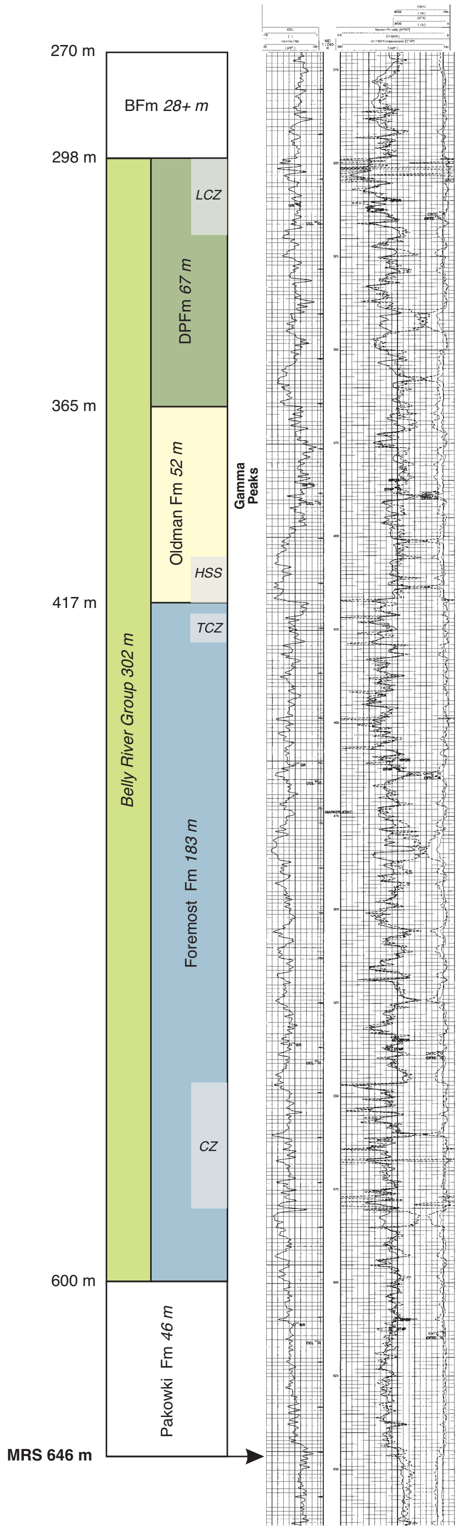

#53
Reference well
08-34-21-20W4

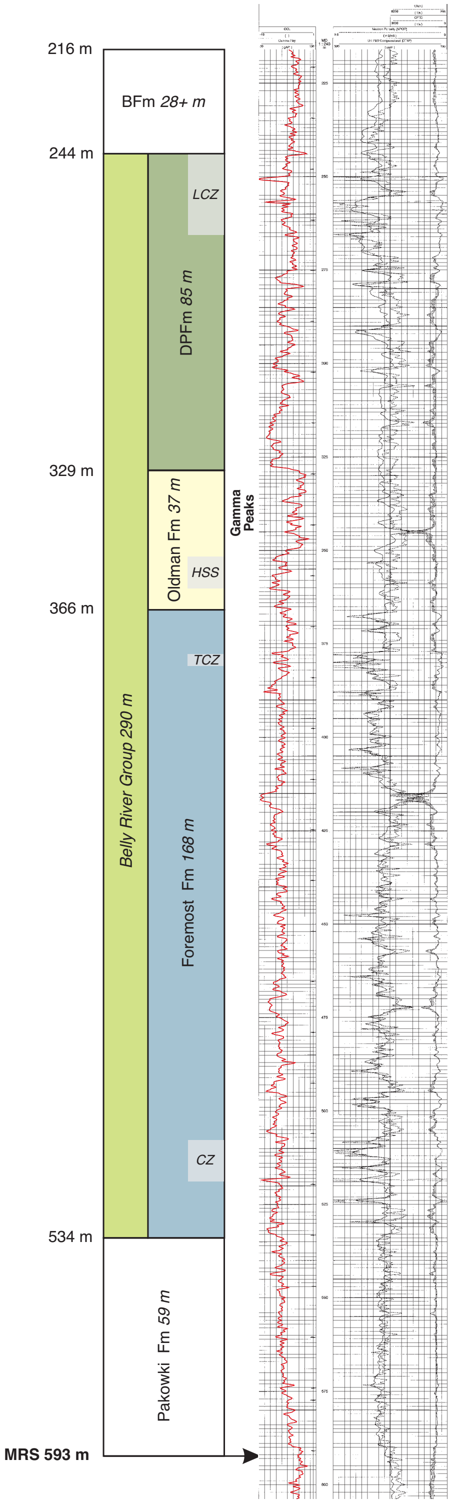

#83
Reference well
15-15-25-20W4

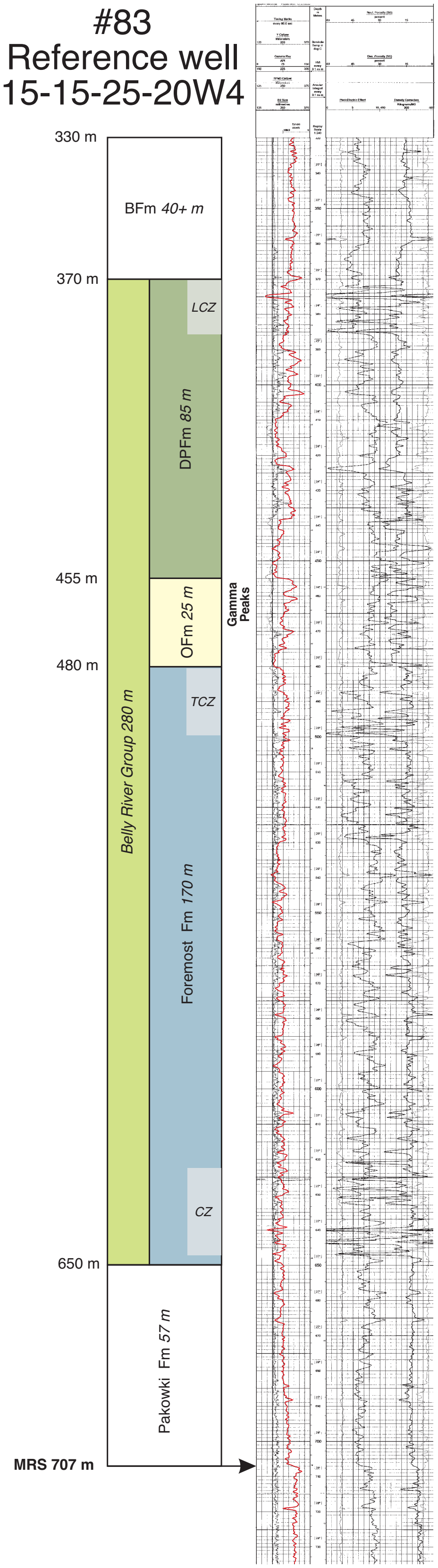

#84
09-04-30-20W4

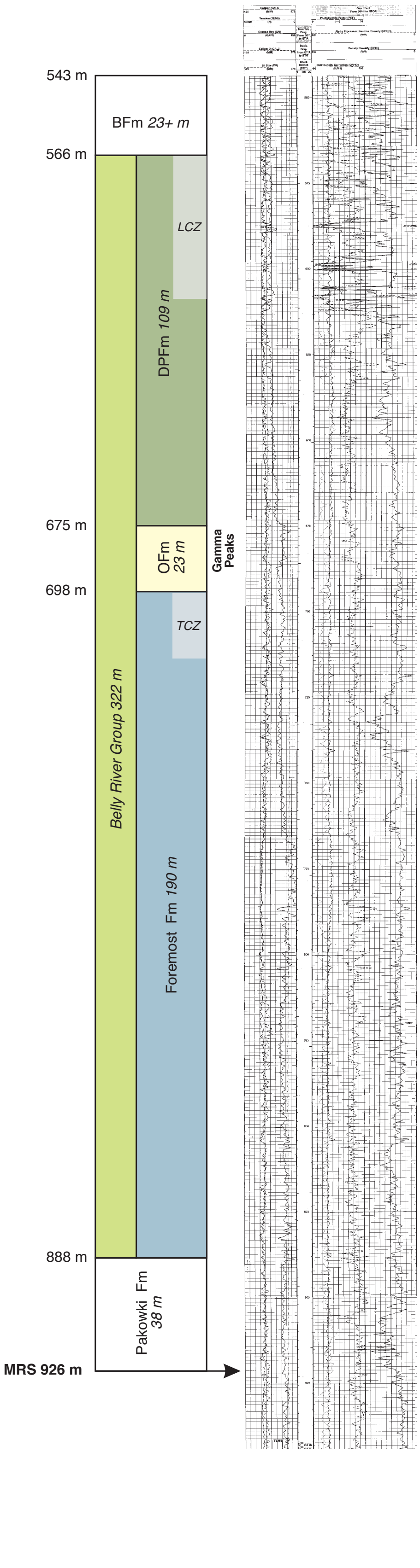

#85
15-27-35-21W4

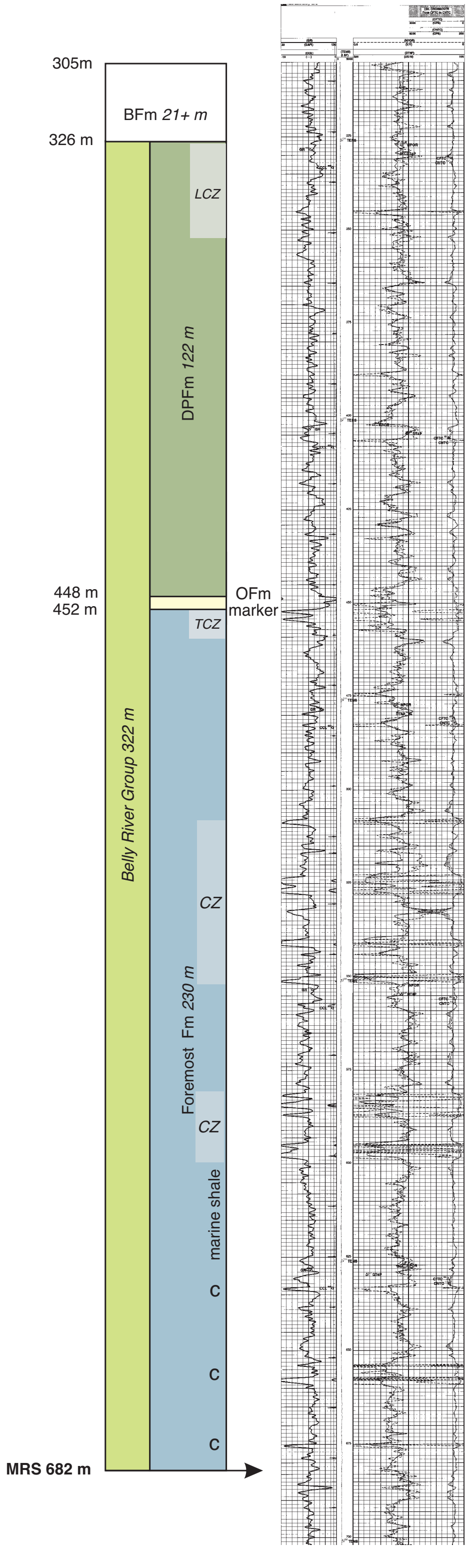

Supplement: S3 Fig — (PDF) [file pone.0292318.s003.pdf]
