# Supplementary figures and images for: Stratigraphic architecture of the Belly River Group (Campanian, Cretaceous) in the plains of southern Alberta: Revisions and updates to an existing model and implications for correlating dinosaur-rich strata
Source: PLoS One. 2024 Jan 25;19(1):e0292318. doi: 10.1371/journal.pone.0292318 (PMC10810474; doi:10.1371/journal.pone.0292318)

#86

16-20-10-03W4

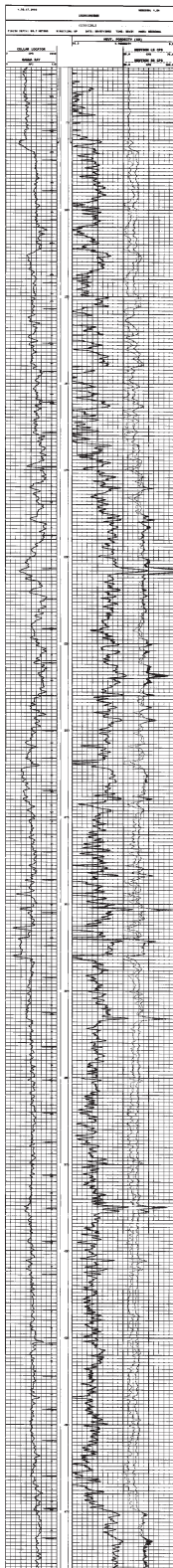

#87

06-34-10-03W4

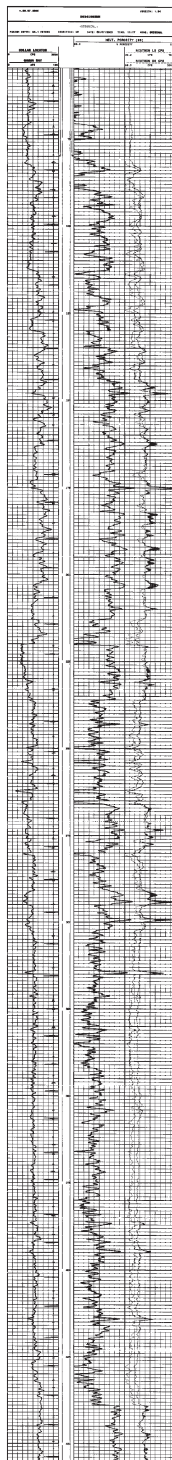

#88

07-14-10-02W4

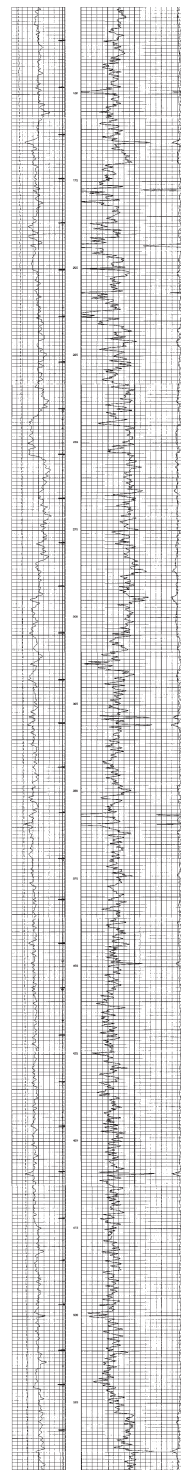

#89

11-29-10-02W4

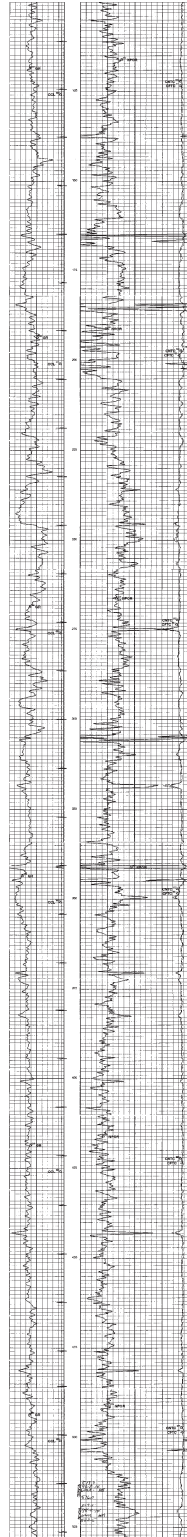

#90

06-36-10-02W4

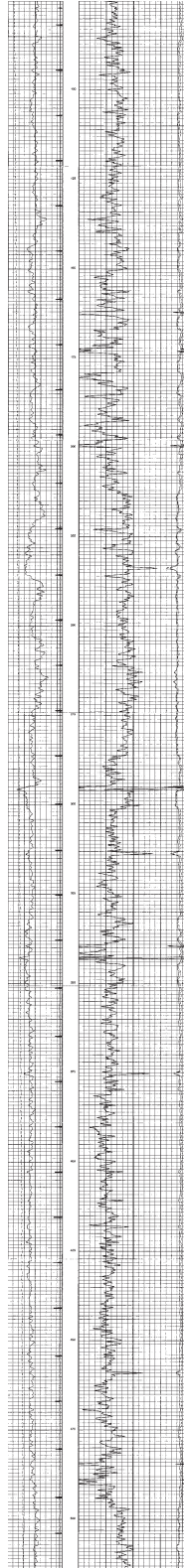

#91

06-06-10-01W4

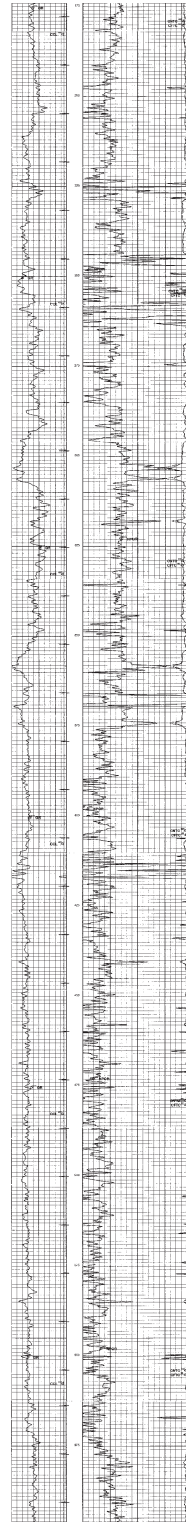

#92

11-29-10-01W4

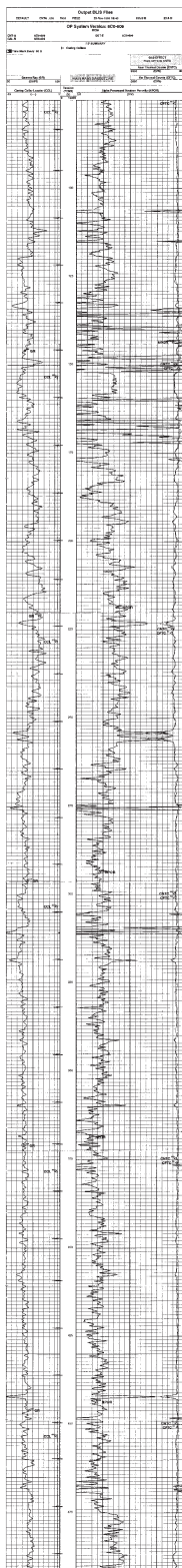

#93

06-22-10-01W4

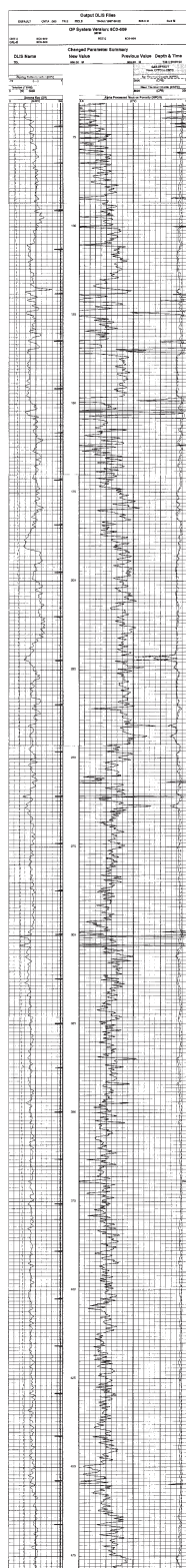

#94

10-11-10-01W4

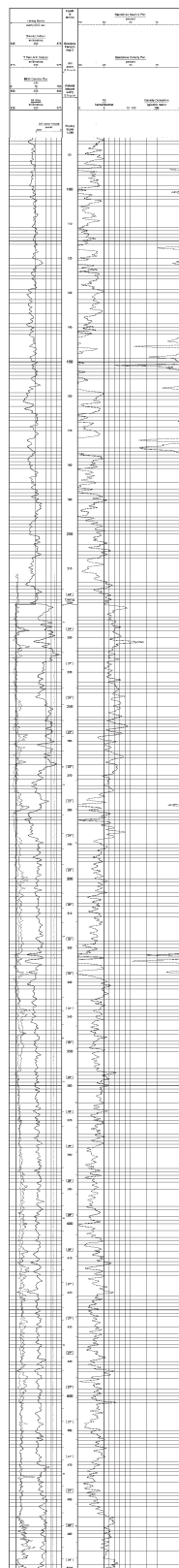

#95

06-02-10-01W4

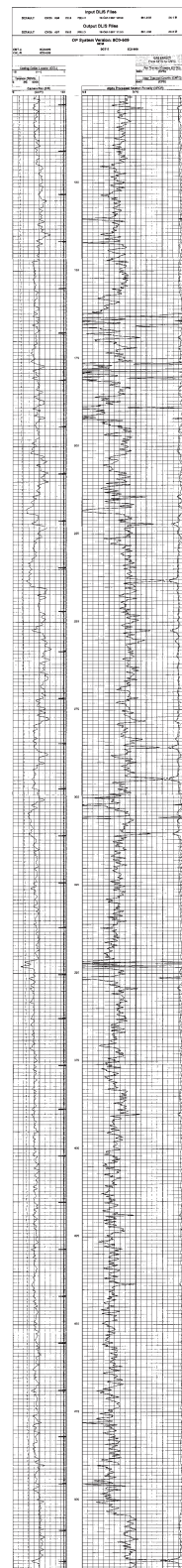

#86

16-20-10-03W4

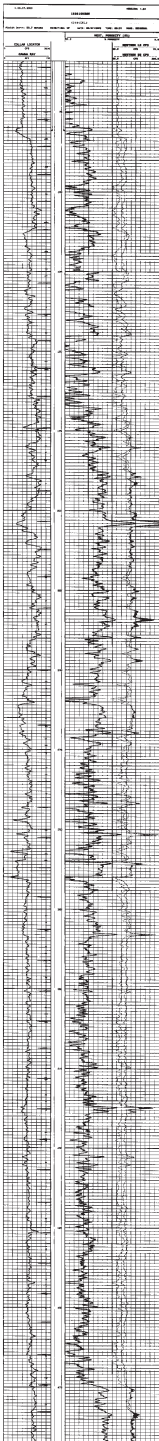

#96

06-18-10-03W4

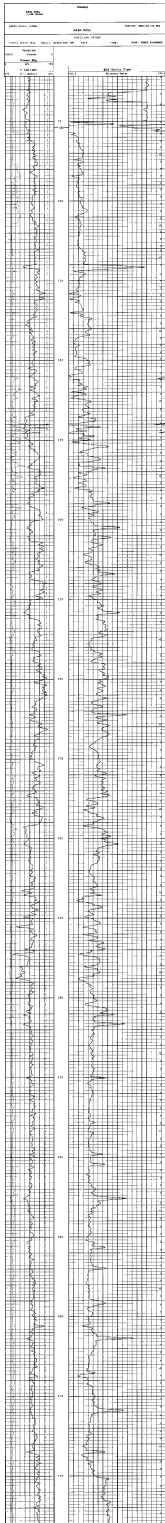

#10

06-06-10-03W4

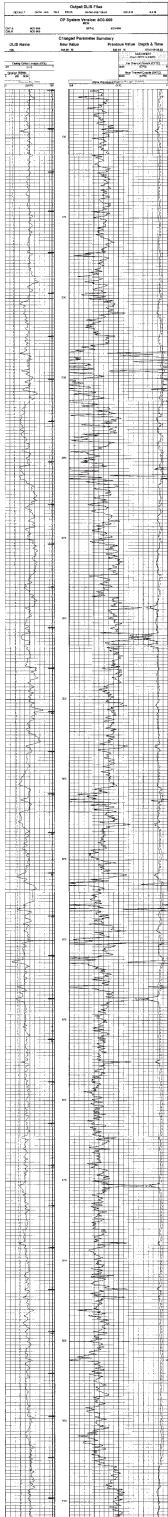

#97

06-36-09-04w4

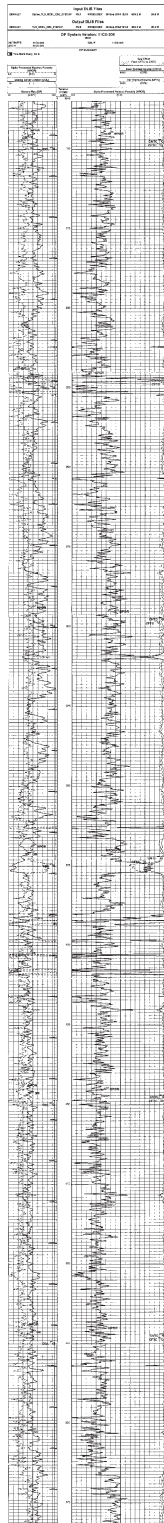

#98

13-25-09-04w4

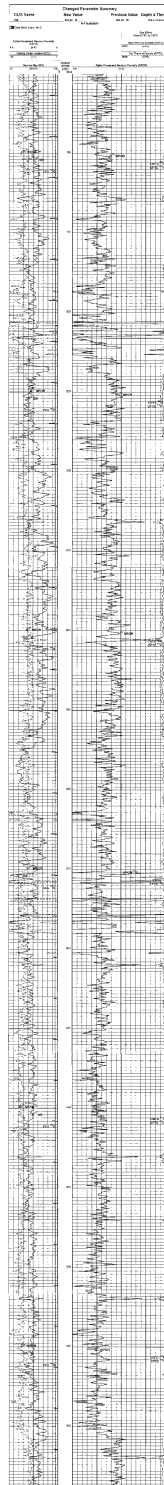

#99

01-23-09-04w4

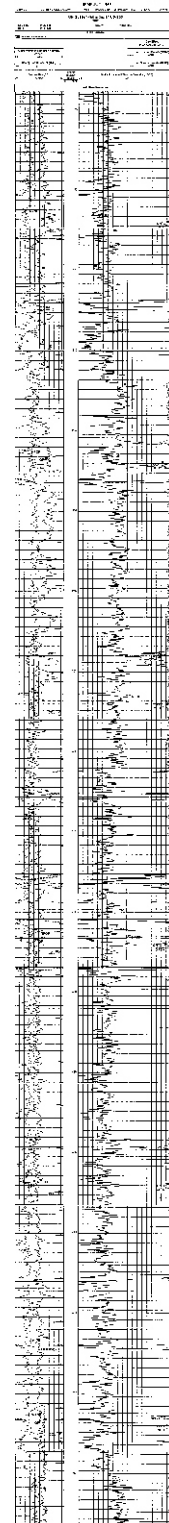

#100

04-14-09-04w4

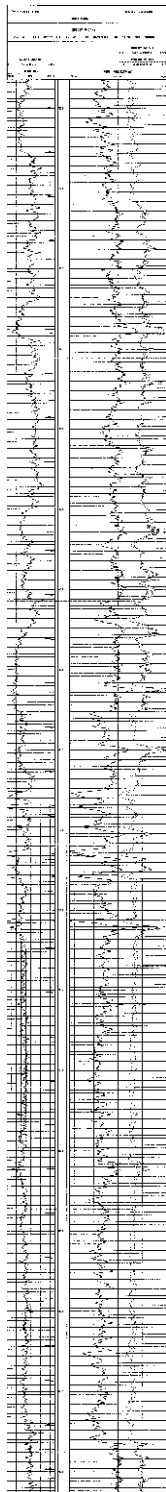

#101

06-02-09-04w4

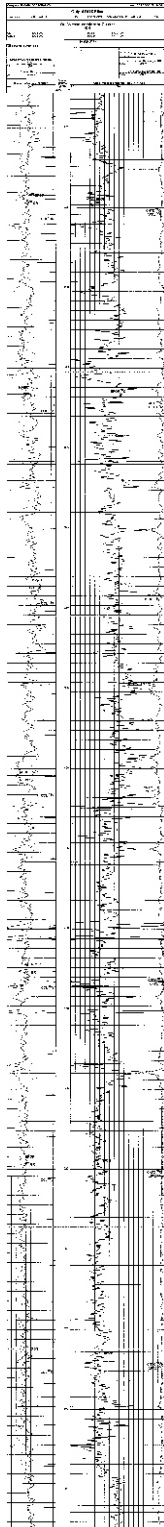

#102

15-35-08-04w4

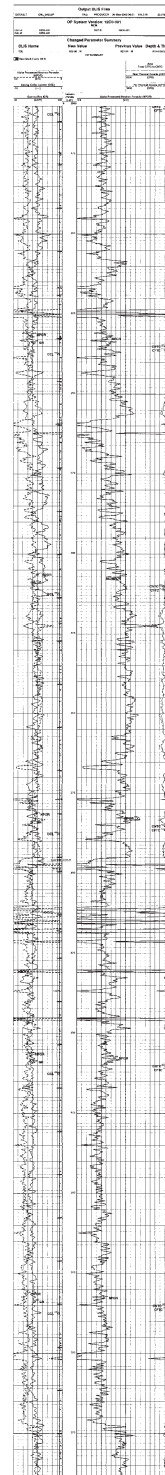

09-26-08-04W4

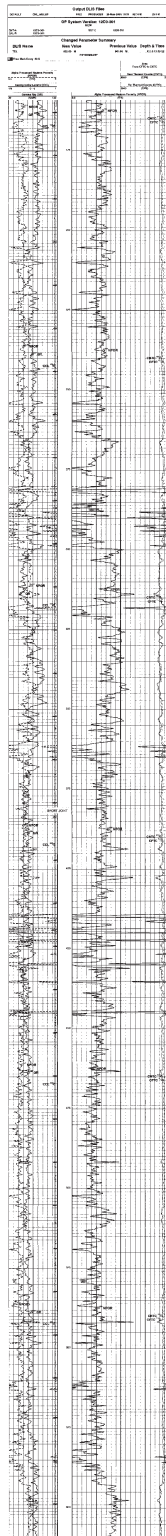

11-23-08-04W4

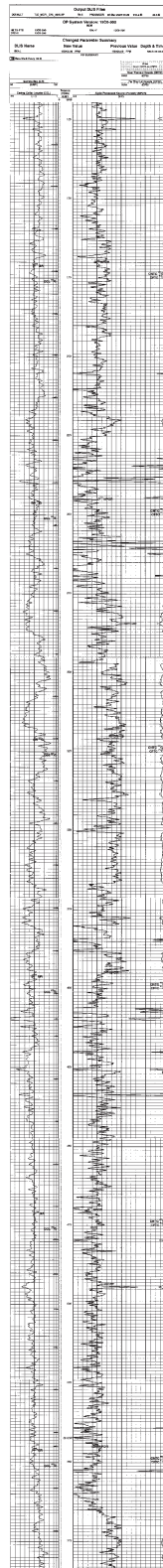

16-12-08-04w4

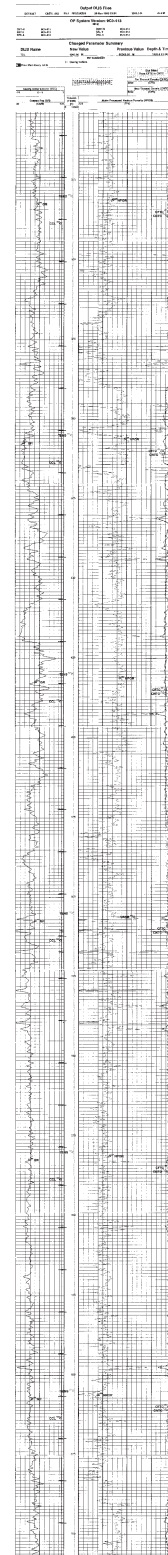

Supplement: S4 Fig — (PDF) [file pone.0292318.s004.pdf]
