# Supplementary material for: Stratigraphic architecture of the Belly River Group (Campanian, Cretaceous) in the plains of southern Alberta: Revisions and updates to an existing model and implications for correlating dinosaur-rich strata
Source: PLoS One. 2024 Jan 25;19(1):e0292318. doi: 10.1371/journal.pone.0292318 (PMC10810474; doi:10.1371/journal.pone.0292318)
Supplement: Attachment — Submitted filename: Response to Reviewers.docx [file pone.0292318.s005.docx]

**Response to Reviewers**

**Academic Editor review**

1. I have revised the ms formatting and file names to bring them in line with PLOS style. I did use PACE to convert from my PDFs and check the figures. However, I found that the quality of the TIFFs created by that program was much poorer than the PDFs and original CorelDraw images. I custom transformed the images to TIFFs myself and was much happier with the results (although they are still somewhat degraded). I strongly recommend that PLOS consider allowing PDFs as text figures (along w TIFF and EPS). PDFs are industry standard and EPS files are somewhat unreliable and are being phased out in the graphics community. TIFFs tend to be pixelated due to the transformation algorithm. Thanks for considering the request.
2. There are no specimens — rock, fossil, or otherwise — used in this study. Thus, further information is not required. No field permits were required at the time that field work was conducted (1980s). Accordingly, I have added a section at the end of the ms that states: “No permits were required for the described study, which complied with all relevant regulations.”
3. I have removed mention of archived field notes in the Methods section. The measured sections that are depicted here (Figs 4–8) are standard sedimentary geology logs that were drafted originally in the field, and thus require no access to field notes for further explanation. Similarly, there are no ethical or legal issues surrounding the data, nor any need to upload written field notes as an additional Supporting Information file.
4. The ms by Eberth, D.A., Evans, D.C., Ramezani, J., Kamo, S., Brown, C.M., Currie, P.J., Braman, D.R, is now published (Canadian Journal of Earth Sciences, 2023). Reference to that publication has been updated throughout the ms.
5. Figure 1 is an original map drafted by me for this ms and contains elements (e.g., Twp-Rg designations, rivers, city locations, my own proposed Belly River outcrop limits, cross-section and well locations) that are either unique to this image or clearly reside in the public domain. In short, Figure 1 is not derived from any one pre-existing image. I replaced figure 2B with a new image that contains new scientific information and was drafted in the same style as other images in this ms.
6. Captions are included for Supporting Information files.
7. The reference list includes no retractions or new references. It does, however, include updated information for the publications by Eberth et al. (2023) and Rogers et al. (2023), which are now published.

**Peer Reviewer review**

The peer reviewer raised only a single issue (#5). The reviewer is curious about the lateral relationships of the Oldman and Dinosaur Park formations to one another. More specifically: should the contact, where it is diachronous, be described and expressed in images as interfingering?

This is an excellent observation and comment, and one that many of us have puzzled over (on and off) during the past 30 years. Well log data clearly document interfingering contacts between marine shales and the Belly River Group (as shown in Figure 2A), but such interfingering has never been clearly observed or documented between the group’s non-marine units where facies differences are much more subtle. Documentation of interfingering would likely require a comparative geochemical analysis of mudstones from many outcrops conducted over many kilometers in order to test for differences in the fine-grained facies of the Oldman and Dinosaur Park formations— an undertaking that would prove expensive and time consuming, and would not be guaranteed to provide important new information.

Whereas it seems entirely reasonable to assume that there should be some degree of mixing and interfingering at the boundaries of these two clastic wedges, we remain hesitant to make that claim or represent it in our figures for fear of misrepresenting the degree to which both wedges are competing at their edges. For example, the subsurface data presented here (e.g., Fig 9, 11) clearly suggest a step-wise relationship between the two wedges, especially at locations along the Alberta-Saskatchewan border. Such step-wise relationships suggest the presence of local areas where the lateral extent of interfingering may be quite limited. Similarly, there are extensive areas between the “steps” where the contact remains more-or-less flat. Together these data suggest that a depiction of interfingering may incorrectly represent the true nature of the interaction of these wedges. I have modified Fig 2A to better highlight the step-like nature of this contact in southeastern Alberta.

In summary, we have opted for use of “shazam” lines in our graphics where we have evidence of interfingering (Foremost-Lea Park and Dinosaur Park-Bearpaw contacts), and avoid their use where such evidence is currently lacking.

To my knowledge there are no other outstanding criticisms of this ms.

David A. Eberth

Senior Research Scientist Emeritus

Royal Tyrrell Museum of Palaeontology

Drumheller, Alberta
